# Chemical Tracking of Temperature by Concurrent Periodic Precipitation Pattern Formation in Polyacrylamide Gels

**DOI:** 10.1021/acsami.1c20640

**Published:** 2022-01-20

**Authors:** Muhammad Turab Ali Khan, Joanna Kwiczak-Yiğitbaşı, Pedram Tootoonchian, Mohammad Morsali, Istvan Lagzi, Bilge Baytekin

**Affiliations:** † Chemistry Department, Bilkent University, Ankara 06800, Turkey; ‡ Department of Physics and BME-MTA Condensed Matter Physics Research Group, Budapest University of Technology and Economics, Budapest H-1111, Hungary; § UNAM, Bilkent University, Ankara 06800, Turkey

**Keywords:** periodic patterns, Liesegang phenomenon, polyacrylamide gel

## Abstract

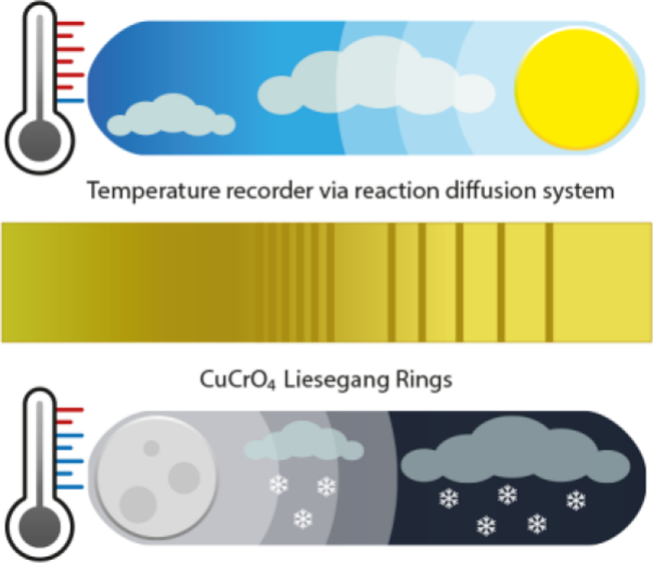

In nature, nonequilibrium systems reflect environmental changes, and these changes are often “recorded” in their solid body as they develop. Periodic precipitation patterns, aka Liesegang patterns (LPs), are visual sums of complex events in nonequilibrium reaction–diffusion processes. Here we aim to achieve an artificial system that “records” the temperature changes in the environment with the concurrent LP formation. We first illustrate the differences in 1-D LPs developing at different temperatures in terms of band spacings, which can demonstrate the time, ramp steepness, and extent of a temperature change. These results are discussed and augmented by a mathematical model. Using scanning electron microscopy, we show that the average size of the CuCrO_4_ precipitate also reflects the temperature changes. Finally, we show that these changes can also be “recorded” in the 2-D and 3-D LPs, which can have applications in long-term temperature tracking and complex soft material design.

## Introduction

**Figure 1:**
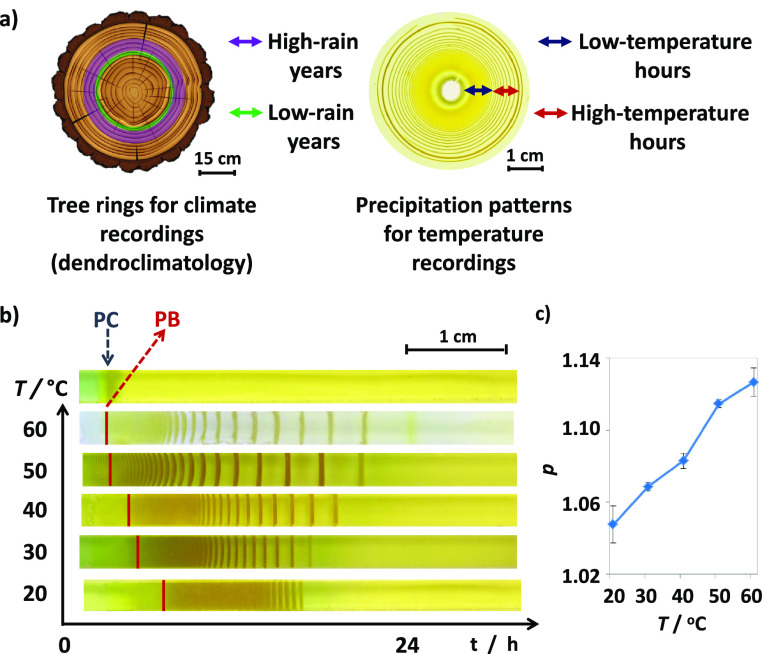
Temperature tracking and recording. (a) LP formation can be used to record and track back the extent and duration of temperature change similar to the recording of low- and high-rain years in the tree ring panel (dendroclimatology). Shown here, the CuCrO_4_ LPs in polyacrylamide (PAm) gels. (see Figure 5a for the details on *T* changes) (b) From top to bottom: CuCrO_4_ LP bands formed in PAm hydrogels at 60, 50, 40, 30, and 20 °C. 1.0 M CuCl_2_ solution applied at the point of contact (PC) is let to diffuse (from left to right in the photos) into gel strips containing 0.01 M K_2_CrO_4_. CuCrO_4_ precipitation begins at points labeled as PB. With every 10 °C increase in temperature, wider “depletion zones” between the precipitation bands, further PB, and further-reaching LPs are observed. (c) Increase in LPs’ spacing coefficient (*p*) with increasing temperature. PC is taken as the reference point for calculations. Error bars in (c) correspond to standard deviations from five independent experiments. See Experimental Section for calculation details.

Patterns found in nature are all unique.^1^ When examined closer, for example, in the periodic patterns of rocks, no two are the same. There are always some differences between the forms we encounter. These differences result from the varying external and internal factors that affect the formation of the patterns. To reveal the mechanisms behind the pattern formation and to describe the pattern formation by mathematical models, synthetic nonequilibrium chemical systems have been employed.^2,3^ An important class of pattern-forming systems known as Liesegang patterns (LPs), discovered more than a century ago by Raphael Eduard Liesegang,^4^ are periodic precipitation patterns resulting from the reaction–diffusion processes.^5^ Typically, the patterns are formed in a hydrogel medium where the diffusion of one reactant is directed in the other, in one dimension (1-D), in two (2-D), or three dimensions (3-D). The precipitation product appears as differently spaced consecutive zones.^6^ The positions of the zones with respect to each other are mathematically explained by the “spacing coefficient”. (Spacing law is one of the benchmark characterizations of LPs and is represented as follows: *p* = *X*_(*n*+1)_/*X_n_*, where *X*_(*n*+1)_ and *X_n_* are the positions of the two consecutive zones measured from the gel/outer electrolyte interface, respectively, and *p* is the so-called spacing coefficient.) There have been studies displaying the effects of electric,^7^ and magnetic fields on LPs.^8^ Some other studies demonstrated the effect of the initial concentrations of the reactants on the pattern morphology.^9^ The gel concentrations and the degree of cross-linking in gels also affect the final LP appearances.^10,11^ There are various studies describing some trends and emergence of new shapes, e.g., helical patterns, at different temperatures.^12,13,22,14−21^ However, these studies were mostly conducted with agarose and gelatin gels that melt above 45 and 30 °C, respectively.^19^ Therefore in the LP-forming systems, the temperature is usually chosen as room temperature.^20^ Nevertheless, among all other factors, temperature is the only factor that affects reaction rates, diffusion coefficients, and precipitation thresholds altogether. In numerical simulations, Antal et al. suggested that a temperature change can cause revert banding, equidistant banding, and it could be used for pattern control of LPs.^23^ However, on the experimental side, there is no study on the temperature field change on such control or monitoring the environmental changes by “reading” a precipitation pattern system. In our previous study,^24^ we illustrated how mechanical deformation could be tracked based on the evolution of the periodic precipitates. This technique was employed to understand the extent of deformation, and the time and duration of the deformation were revealed in the visual appearance of LPs. In this study, we investigate the dynamics of pattern formation under varying temperatures. We show that a developing LP can visually mark the extent and duration of the environmental temperature change (Figure 1a), imitating the “dendroclimatology” (the response of the tree ring growth to the climate) in a synthetic system as described below.

## Results and Discussion

LPs can form in various porous media.^25−28^ Our choice was PAm hydrogel since this is a covalently cross-linked hydrogel with a glass transition temperature above 190 °C.^29^ This gel provides a homogeneous medium for the formation of CuCrO_4_ patterns, as we have reported before.^24^ To demonstrate the effect of the temperature on the formation of LPs in a common LP system, we prepared gels with 0.01 M potassium chromate as the first reactant (or “inner electrolyte”) in rectangular plexiglass molds (Figures S1–S3). We then introduced 1.0 M copper(II) chloride as the second reactant (or “outer electrolyte”) to the gel column from one side of the rectangular mold. The mold was thermostated at a constant temperature for 24 h. (The concentrations of the crosslinker, outer and inner electrolyte were previously optimized according to the best LPs appearances as presented in Figure S4.) This system is called “1-D” since the diffusion is in one direction, specifically from left to right in the photo of the gels shown in Figure 1b. The changes in the LPs forming at constant temperatures, 20–60 °C, are shown in the photos of 1-D gel strips in Figure 1b. From the photos, it is clear that with every 10 °C increase in temperature, (1) wider “depletion zones” (the gel regions with no significant precipitation) between the precipitation bands occurred, (2) the position of the last band formed in 24 h was further from “the PC” of the outer electrode, and (3) the “precipitation begin” was further from the PC at higher temperatures. The patterns were then analyzed by gray value analyses (Figure S5), which gives precise values of band spacings and helps to calculate the spacing coefficient (*p*) values. For all LPs in the samples, the *p* values were computed, taking PC as a reference point for all the samples (Figure S5). As shown in Figure 1c, the spacing coefficient increases with the temperature increase. The spacing coefficient and the other visual information implicit in each of these LPs can also be detailed, modeled, and augmented by calculations (Figure S6), which give a precise quantitative explanation of the systems. This precision encouraged us to make a system that can reflect and record the changes in the environmental temperature as described below.

**Figure 2:**
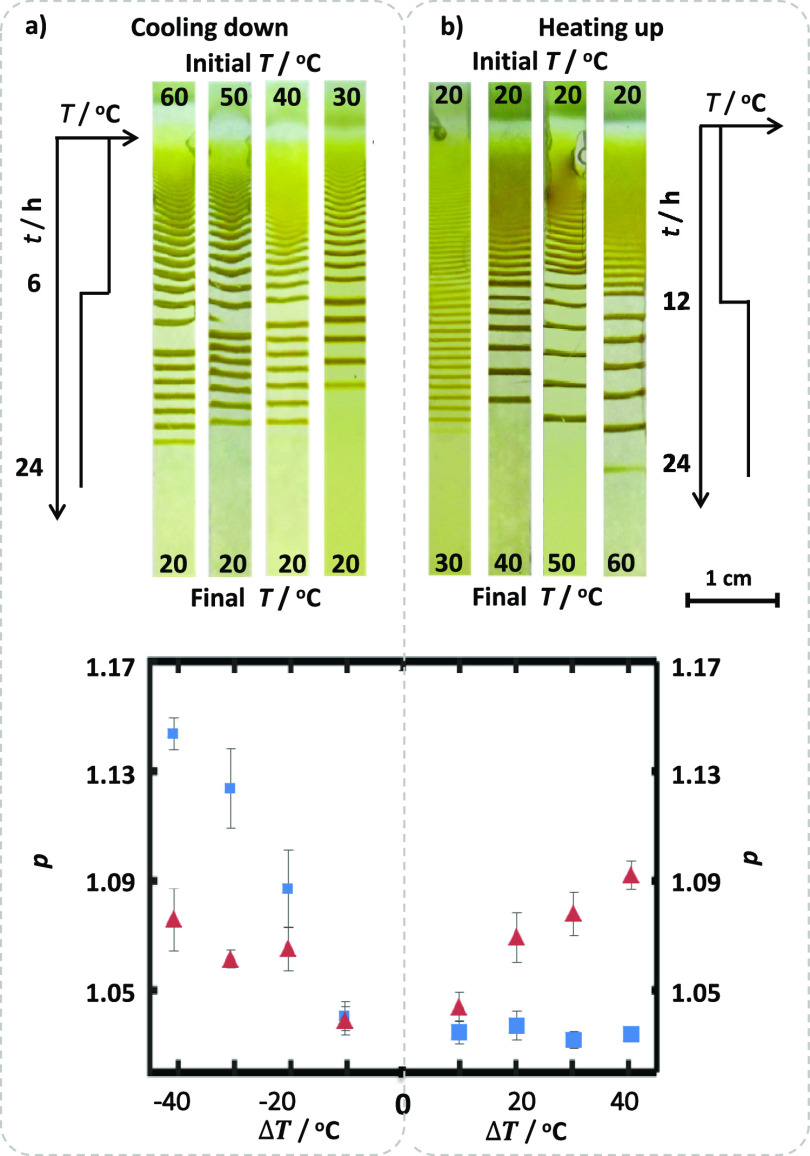
LPs can “record” the cooling or heating of the environment as they develop. (Top) Gel samples (a) placed at *high* temperatures (30, 40, 50, and 60 °C) for 6 h are then *cooled down* to 20 °C; and (b) placed at *low* initial temperature (20 °C) for 12 h are then *heated up* to 30, 40, 50, and 60 °C, during the formation of the CuCrO_4_ LP bands. Temperature transitions are visible in the abrupt changes in the spacings between the bands. (Bottom) Calculated pretransition (squares) and post-transition (triangles) spacing coefficients of the LPs. The extent of the corresponding temperature change is reflected in the difference between the pre- and post-transition spacing coefficients. For samples in (a) and (b), 1.0 M CuCl_2_ solution is let to diffuse (from top to bottom in the photos) into gel strips containing 0.01 M K_2_CrO_4_. Error bars in (a, b) correspond to standard deviations from five independent experiments. See the Experimental Section for calculation details.

To show the effect of a changing temperature field on concurrently forming LPs, which would be a sign that these systems can be used to provide information about the changing environmental temperature, we performed two sets of experiments, cooling and heating (Figure 2a,b, respectively) the LP-forming system during the LP formation. In the “cooling down” experiments, we let the four LP-forming samples at initial fixed temperatures ranging from 30 to 60 °C (Figure 2a). Then at the 6th hour of the pattern formation, after a considerable number of LP bands had formed and the spacing coefficient (red triangle marks) were calculated, the samples were cooled down to 20 °C with the ramp 10 °C/min creating temperature differences (Δ*T*) of −10, −20, −30, and −40 °C. The spacing coefficient decreased, and this decrease was proportional to the amount of Δ*T* applied. On the other hand, in the “heating” experiments, all four samples were placed at 20 °C initially (Figure 2b). After 12 h, the samples were heated up to the final temperatures of 30, 40, 50, and 60 °C creating temperature differences (Δ*T*) of +10, +20, +30, and +40 °C, respectively. The increased temperature increased the spacing coefficient of LPs, and the amount of this increase was again proportional to the Δ*T*. The results of both experiments show that LPs evolved visually and mathematically (Figure S7) distinctive from each other in terms of the extent of the temperature change (Δ*T*). It should be noted that the spacing coefficient of the pattern after cooling the sample to 20 °C is higher in comparison to the one developed at fixed 20 °C. Similarly, the spacing coefficient at fixed 60 °C is higher in comparison to the one developed after heating the sample to 60 °C. The reason for this behavior is the depletion of copper ions. As the diffusion front of the copper ions proceeds inside of the gel medium, its concentration is diminishing because of the formation of copper chromate precipitate. This effect is even more pronounced at higher temperatures, when the solubility of copper chromate increases, meaning that even more copper ions are necessary for the band to form. To support our claim, we calculated the spacing coefficient values of LPs formed with 0.1, 0.5, or 1.0 M CuCl_2_ solution as the outer electrolyte (Figure S8). It can be seen that when the concentration of copper ions decreases, the spacing coefficient of the pattern increases. The heating and cooling experiments were repeated for various transition times (Figure S9) and transition rates (Figure S10). Each experiment resulted in a visually distinct and unique LP, recording the nature of the temperature change in the environment.

Why do the patterns forming at different temperatures have different spacing coefficients (Figure 1c), and why is the pattern formation affected by a temperature change (Figure 2)? As we have mentioned, the temperature is the only factor that affects reaction rates, diffusion coefficients, and precipitation thresholds altogether. Therefore, the formation of visually distinct patterns with temperature can be explained by the influence of temperature on (1) diffusion of copper ions, (2) the rate of formation of copper(II) chromate, and (3) the solubility product of copper(II) chromate. In Figures S11 and S12, the diffusion of copper ions in PAm gel and the rate of copper(II) chromate formation are shown to increase with increasing temperature. (The changes in the reaction rate are determined with counter-diffusion experiments (Figure S13)). The increase in the reaction rate and the diffusion coefficient alter the time evolution of LPs. The space–time (time law) relationship of LPs is shown in Figure S14. From this relation, we see that LPs form further in the spatial coordinate at a faster rate at higher temperatures, as displayed in the experiments (Figures 1 and 2). The third point, the effect of temperature on the solubility product, is also of great importance: the Liesegang phenomenon is observed for sparingly soluble salts. Periodic bands of the salt arise inside hydrogels when colloids aggregate to form bigger particles. This process occurs when the concentration of colloids at a certain point in the gel (band location) surpasses the precipitation threshold. Therefore, the solubility product plays a vital role in determining a precipitation threshold for the system.^16,30^ The direct relationship between precipitation threshold and solubility product indicates that salts with high *K*_sp_ will require a higher concentration of colloids to aggregate and vice versa. This relationship also alters the spacing between the bands in the following way: As a reaction–diffusion system propagates, the concentration of inner electrolyte depletes behind and in front of the band. The outer electrolyte diffuses further, and the concentration of colloids rises to form the next band, however this time at a place further from PC in the hydrogel.^30^ We determined that the solubility product of CuCrO_4_ increases with increasing temperature, as shown in Figure S15. Therefore, at higher temperatures, the creation of a precipitation band will require a higher concentration of the forming colloids. The colloids will also aggregate further from PC in the spatial coordinate to form the next consecutive band. Therefore, at higher temperatures, the spacings between patterns formed are higher, as observed in Figures 1 and 2. (For a more detailed discussion on the relationship between reaction rate coefficient (*k*_p_), diffusion coefficient (*k*_d_), and precipitation threshold (*K*_sp_) and pushed and pulled fronts, see Supporting Information).

**Figure 3:**
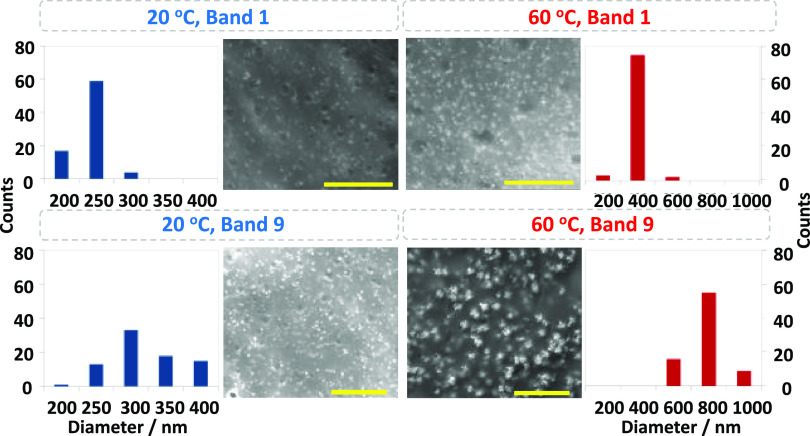
Scanning electron microscopy (SEM) micrographs and size histograms of the CuCrO_4_ precipitates in LP bands at different band numbers and temperatures. With increasing LP band number (Bands 1–9) and temperature (20–60 °C), the average particle sizes increase. Blue and red bars in the histogram are the particle sizes obtained at 20 and 60 °C, respectively. SEM samples were prepared from the LP bands shown in Figure 2. Scale bar is 5 μm. ImageJ software is used to generate the histograms. For complete sets of SEM images of LP bands of Figure 2, particle size histograms see Figures S16 and S17.

When the bands of the LPs are investigated by SEM, the interplay of the processes mentioned above (reaction–diffusion-solubility) can also be visualized at the micro-level. In the literature, it is stated that the size of the colloids rises in each consecutive band in an LP.^5,31−34^ This particle size increase can also be observed in the CuCrO_4_ LP bands formed at a fixed temperature (Figure 3). For example, for 20 °C, the LP Band 1 has smaller particles than the Band 9 forming at the same temperature. In addition to this change in size with band number, we can also expect different particle sizes for the same generation of bands forming at different temperatures. At lower temperatures, the *K*_sp_ values are lower, the supersaturation is high, and the colloids surpass the precipitation threshold is easier, leading to smaller particle sizes. As shown in Figure 3, bands 1 and 9 formed at 60 °C have larger particles than the same bands formed at 20 °C. (For SEM micrographs of all precipitates in LP bands 1 to 9 for temperatures 20, 30, 40, 50, and 60 °C, see Figures S16 and S17). We must also state that the SEM micrographs of the nonband areas revealed no precipitate (Figure S18). This observation was also supported by EDX spectra taken from the band and nonband regions, showing Cu and Cr peak at 8.0 and 5.4 eV in the former, and only Cu peak at 8.0 eV in the latter (Figure S18).

**Figure 4:**
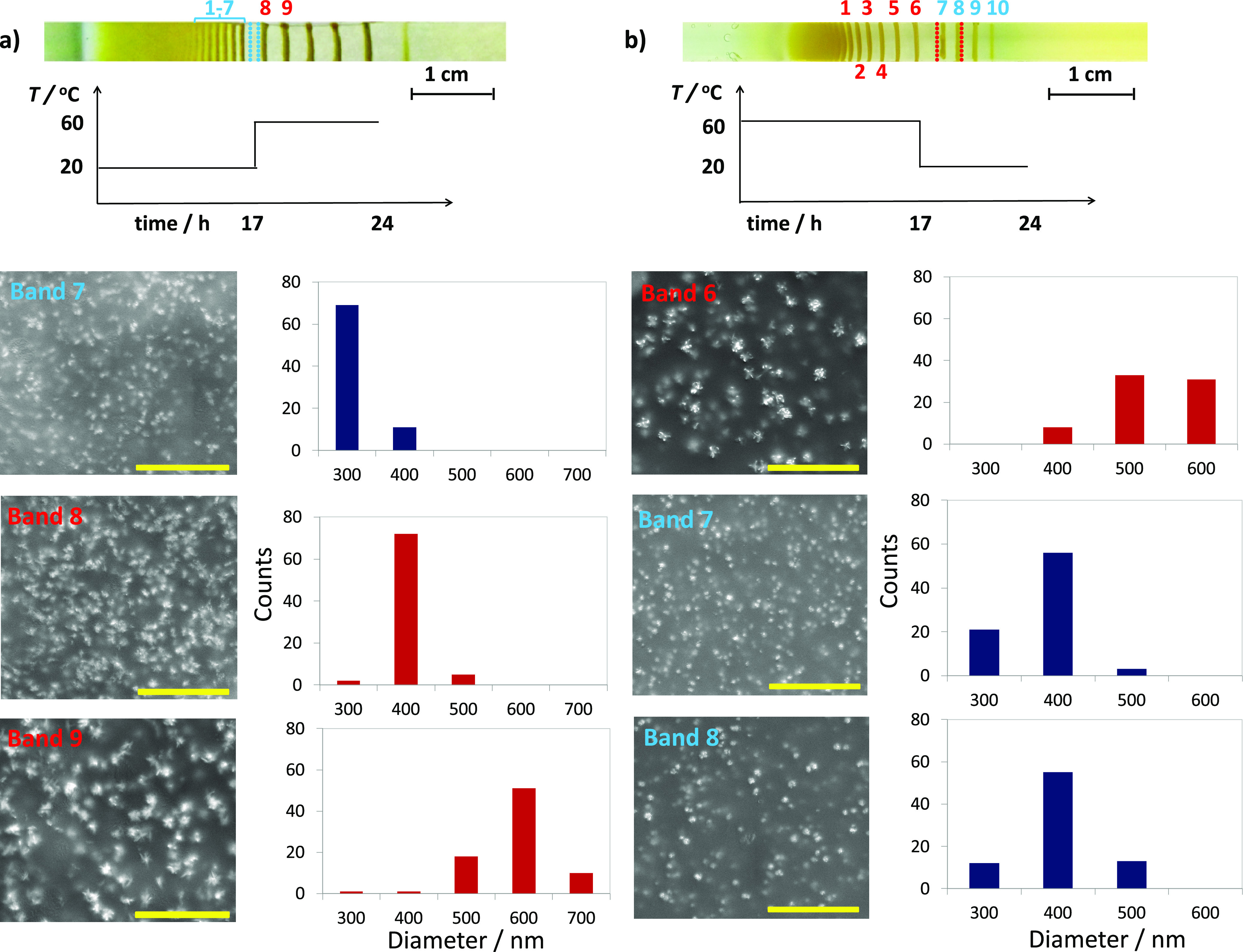
Microscopic changes in the LP precipitates display the cooling or heating of the environment during the LP formation. Gel sample (a) placed at a lower temperature (20 °C) for 17 h is then heated up to 60 °C (blue dashed lines show the position of the consecutive bands if the temperature were kept constant at 20 °C), (b) placed at higher initial temperature (60 °C) for 17 h are then cooled down to 20 °C (red dashed lines show the position of the consecutive bands if the temperature were kept constant at 60 °C), during the formation of the CuCrO_4_ LP bands. Temperature transitions are visible in the average particle size changes of the consecutive bands near the corresponding SEM micrographs of the LP bands with given numbers. The extent of the temperature change is reflected in the differences between the pre- and post-transition spacing coefficients. For samples in (a) and (b), 1.0 M CuCl_2_ solution is let to diffuse (from left to right in the photos) into gel strips containing 0.01 M K_2_CrO_4_. Scale bar is 5 μm. See the Experimental Section for other details.

The temperature not only controls the macroscopic spacings of the bands (Figures 1 and 2) but also the sizes of the precipitated particles (Figure 3), providing additional information emerging at the micro-level. Previously, we saw that the changes in temperature during the LP formation affect the band spacings at the macro level, “recording” these changes “in the bands”. We wondered if such information is also translated in the particle sizes at the micro-level. Therefore, we investigated the SEM micrographs of the LPs forming under an increasing or decreasing temperature. For the first case, the LPs are initially let to develop at 20 °C until the completion of the 7th band. Then the temperature was raised to 60 °C in which the consecutive bands formed. As expected, since the band number increases, the 8th and the 9th bands have larger particles than the 7th band since they have a larger band number. However, in this case, the increase is even more pronounced in comparison to the LPs forming at a fixed temperature, since in the meantime, there was an increase in the medium temperature. For example, the average sizes of the particles in Band 9 increased about twice the average size of the ones in Band 6. (At 20 °C only, this ratio is just 1.2) (see Figure S19 for a complete analysis). More interestingly, the particle sizes of the precipitates at higher band generations decrease when the system’s temperature is lowered during the formation of LPs (Figure 4b). This “reversing” of the particle size is visible as large bright spots in the SEM micrograph of the Band 6 (with an average particle size of 480 nm), which is the last band formed at the initial temperature of 60 °C, in comparison to the small spots (with an average of 380 nm) in the SEM image of the zone 7, the first band formed at 20 °C. (Figure S20).

As shown above, the extent of the temperature change (Δ*T*) (of tens of °Cs) is well-visible on the LPs. The smallest Δ*T* value we can probe with these recorder LPs is 2.0 °C. Of course, this can be improved if the gels can be prepared more homogeneously by special techniques, which we have not attempted. Next, we have also probed the effect of the Δ*T* ramp rate on the “relaxation” time of the system. This relaxation can be termed as the sensitivity of the system to the rate of temperature changes. In the experiments, using 0.1, 1.0, and 10 °C/min rates, we observed that this sensitivity is around 1.0 °C/min, where expected adaptation of the LPs to the change was observed as the fast decrease in the band spacings but 10 °C/min was too fast to detect the change with the LP temperature recording. (For a detailed discussion, see Figure S21 (cooling ramps), Figure S22 (heating ramps), and the related Supporting Information text.)

**Figure 5:**
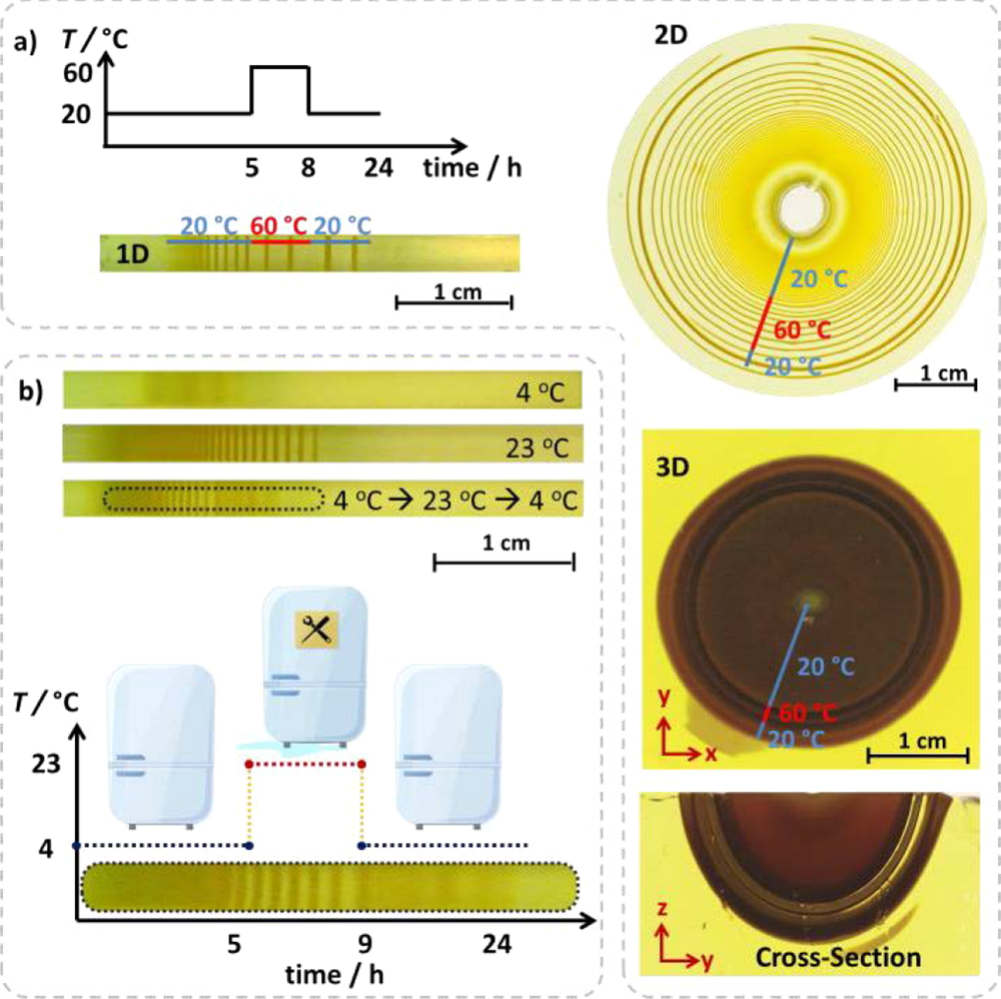
Tracking the environmental temperature by (a) 1-D, 2-D, and 3-D LPs. Gel samples placed at 20 °C for 5 h are then heated up to 60 °C for 3 h, then cooled to 20 °C. Temperature transitions are visualized in the abrupt changes in the spacings between the bands. (b) Refrigerator that did not work overnight between the 5th hour and 9th hour can be spotted by the changes in the LP developing in the fridge during the night.

The 1-D diffusion experiments can easily be adapted to 2-D or 3-D diffusion systems (Figure 5a). In our experiments, the 2-D reaction–diffusion systems were prepared in a mold presented in Figure S2 with a gel thickness of only 2 mm, which allows the diffusion of the Cu^2+^ ions from the stamp placed at the center, essentially only on the 2-D plane. The 3-D system has the same setup in a container of 3.8 cm depth; therefore, a thicker 3-D gel is formed. Cu^2+^ ions from the stamp placed at the center of the gel can diffuse to all three dimensions. In all these three configurations, we performed the same experiment: We let the diffusions of Cu^2+^ ions at 20 °C for 5 h, then ramped the temperature to 60 °C for 3 h, and then cooled the whole system down to 20 °C again. The 2-D example (also shown in Figure 1a) displayed a similar behavior as 1-D with respect to the temperature changes. The final 2-D pattern also looked like the tree rings displaying the recorded changes in the rainy and dry climates. The spacings between the LP rings increased upon an increase in the temperature; narrower depletion zones are observed upon successive lowering of the temperature. In 3D (Figure 5a), the cross-sectional view represents a cut in the middle of the 3D gel block along the added *z*-axis. Again, the zone for an increase in temperature is visible with wider spacings (lighter areas), and the decrease in temperature was visible as semispheres getting closer to each other, forming darker zones.

## Conclusions

In summary, the pattern evolution and the product particle sizes are affected by the magnitude, time, and steepness of the temperature step taken during the LP formation. This thermal information can be recorded within the bands, rings, and semispheres in all three dimensions. Although we have shown that the events leading to these changes are quite complex, we could identify the chemical processes and display how the patterns are “written” by concurrent temperature changes. The visual appearance of the patterns can be used to track the temperature changes, making LPs suitable for long-term (with this CuCrO_4_ system, a couple of hours of) environmental temperature tracking. We displayed this in an example in Figure 5b, where we show the difference between the patterns formed in a fridge that worked overnight without any problems (top gel, 4 °C) and a fridge that was not working for 4 h during the night but was working again when we came to the lab in the morning (bottom gel, 4 °C → 23 °C → 4 °C). We believe that these trackers with LPs can help for a straightforward recording of temperature in such cases when, e.g., a precious cargo is needed to be cooled during a transfer or as a recorder of the temperature of the soft material such as in soft robotics. In addition to this, our proposed method can be used to track and analyze the environmental changes in geochemical systems in which no other conventional methods can be employed. Periodic patterns can frequently appear in rocks and minerals,^28,35^ as also simulated by Sultan et al.^36,37^ Any deviation from the spacing law (i.e., changing in the spacing coefficient) might indicate that the environmental factors (for example temperature) changed during the pattern formation. From the analysis of the pattern, the extent and the duration of the environmental changes can be retrieved.

We have also shown that a reaction–diffusion model can qualitatively capture the experimentally observed phenomena, namely, the dependence of the spacing coefficient on the temperature and the change of the pattern characteristics as the temperature was changed (increased or decreased) in single experiments. The reaction process was based on a widely used sol-coagulation mechanism incorporating the continuous formation of the intermediate species and threshold-limited coagulation step. The effect of the temperature was incorporated by changing the diffusion coefficients of the chemical species (reactants and intermediate) and the coagulation (precipitation) threshold. The excellent qualitative agreements between the results of the experiments and numerical simulations suggest that the main governing factor is the interplay of the changed diffusion coefficients and precipitation threshold induced by the temperature change. A deeper understanding of this interplay could provide new routes to design and engineer various types of periodic precipitation patterns such as equidistant, revert, and irregular patterns. Finally, in general, we showed that temperature provides an additional degree of freedom for material design in different geometries through reaction–diffusion, opening new pathways in complex material design.

## Experimental Section

### Chemicals and Reagents

Acrylamide (AA), *N,N′*-methylene(*bis*)acrylamide (BIS), potassium peroxydisulfate (KPS), *N,N,N′,N′*-tetramethylethylene-diamine (TEMED), and methanol were purchased from Sigma-Aldrich. Potassium chromate and copper(II) chloride dihydrate were purchased from Merck. Ecoflex 0030 was purchased from Smooth-On.

#### Preparation of Molds for 1-D, 2-D, and 3-D Gels

Molds for 1-D, 2-D, 3-D and counter-diffusion experiments were designed in Adobe Illustrator and made from plexiglass using a laser cutter. Two millimeter thick Plexiglas sheet was used for all experiments, thus the thickness of the gels in 1D, 2D and counter-diffusion experiments was 2 mm as well.

#### AA Hydrogel Preparation

Gels were prepared by mixing 0.723 g of AA, 0.003 g of BIS, 0.01 g of potassium persulfate, and 0.01 g of potassium chromate as the inner electrolyte. Then, 4.8 mL of water was added to dissolve all the ingredients. The solution was ultrasonicated for 2 min. Ten microliters of TEMED was added to the gel solution. The gel solution was then transferred to the mold and the top of the mold was closed by a piece of Plexiglas. After 24 h, 1.0 M CuCl_2_ was introduced to the top of the gel. A small drop of oil was added on top of CuCl_2_ solution inside the mold to reduce the evaporation of CuCl_2_ solution. The gel was then placed horizontally, onto the home-built device at the desired temperature.

#### Determination of Spacing Coefficient

LPs were quantified by extracting the information about the position of the bands using ImageJ software. Image of LPs was loaded to ImageJ and the Gray value of a single-line selection was plotted. Gray value refers to a combination of the three color components of the image, i.e., red, green, and blue. The formula used for quantifying the gray value was 0.299red + 0.587green + 0.114blue. Gray value plot showed the bands as the sharp dips in the gray value. Following that, the raw data of the gray value plot were loaded into MATLAB. The negative of the gray value was plotted so that bands appeared as the peaks. Position, width, and prominence were determined by a built-in functionality of MATLAB, i.e., Findpeaks. The position of the band (*X_n_*) was determined for each peak, by finding the distance at which local maxima in negative gray value occurred. Prominence was the maximum gray value. Width was determined by full-width at half maxima. The spacing law was determined by taking a logarithm of positions then plotting the graph of ln(*X_n_*) versus the number of bands. The slope of the graph was the spacing coefficient.

#### Modeling

To model the experimentally observed phenomena, we used a sol coagulation.^24,38^ Briefly, the model consists of two reaction steps, the sol formation A(aq) + B(aq) → C(aq), where A, B, and C are the outer electrolyte, the inner electrolyte and the intermediate product (sol) that can transform into precipitate (D), C(aq) → P(s). The reaction (precipitation)-diffusion process can be described the following set of partial differential equations
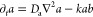
1
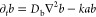
2
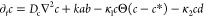
3
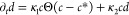
4Here *a*, *b*, and *c* are the concentrations of A, B, and C respectively, while *d* is the density (concentration) of the precipitate, P. *D*_a_, *D*_b_, and *D*_c_ are the diffusion coefficients of the outer electrolyte, inner electrolyte, and intermediate species. *k* is the chemical rate constant for the sol formation, and κ_1_ and κ_2_ are the rate constants for the coagulation and the autocatalytic precipitate formation, respectively. c* is the coagulation threshold concentration. Θ denotes the Heaviside step function.

Reaction–diffusion eqs 1^2^,3,4 were solved using the method of lines technique (finite-difference spatial discretization method using an equidistant grid with the backward Euler method for the integration of the ordinary differential equations). We used the following initial conditions: *a*(*t* = 0, *x*) = 0, *b*(*t* = 0, *x*) = *b*_0_, *c*(*t* = 0, *x*) = 0, *d*(*t* = 0, *x*) = 0. No-flux boundary conditions were applied for all chemical species at both ends of the domain except the outer electrolyte, where a Dirichlet boundary condition (*a*(*x* = 0) = *a*_0_) was used at junction point of the electrolytes, where *a*_0_ is the initial concentration of the outer electrolyte. In the model, we limited the density of the formed precipitate (*d*). If the density of the precipitate at the given space position reaches a threshold (the maximum density of precipitate, ρ), no further growth occurs at the given position, and there is no coagulation threshold at the neighboring positions. This assumption can be mathematically expressed as
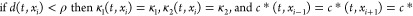
5
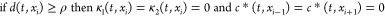
6where *d*(*t*, *x_i_*), κ_1_(*t*, *x_i_*), and κ_2_(*t*, *x_i_*) are the density of the precipitate and reaction rate constants for the precipitation and coagulation at the given grid point (*x_i_*). *x*_*i* – 1_ and *x*_*i* + 1_ denote the neighboring grid points. The following parameter set was used in the numerical simulations: *k =* 1 M^–1^ s^–1^, κ_1_ = 1 s^–1^, κ_2_ = 1 M^–1^ s^–1^, c* = 6 × 10^–3^ M, ρ = 3 × 10^–2^ M, *a*_0_ = 0.5 M, and *b*_0_ = 0.01 M. The time step (Δ*t*) and the initial grid spacing (Δ*d*) were 1 s and 10^–4^ m, respectively.

To incorporate the effect of the temperature in the model, the diffusion coefficients of the chemical species were temperature dependent as *D*_a_ = 9.76286 × 10^–9^ – 9.9662 × 10^–11^*T* + 2.47273 × 10^–13^*T*^2^ m^2^ s^–1^, where *T* is the thermodynamic temperature, and *D*_a_ = *D*_b_ = *D*_c_.^39^ The coagulation threshold concentration was the function of the temperature and varied in the model as c*(*T*) = 0.5 + 5 × 10^–3^ (*T* – *T*_0_), where *T*_0_ was 293 K.

### Tracking Diffusion of Copper Ions and Determination of Reaction Rate’s Dependence on Temperature

The diffusion of copper ions was qualitatively tracked by converting the images from RGB to HSB stack in OpenCV. Following that, a mask was applied for HSV values of [28, 0, 0]. HUE values above 28 were masked by the color filter. The resulting image was converted to a binary image of 0 and 1, where 0 illustrates HUE values below 28 and 1 all the HUE values above 28. In the final image, copper ions were represented as white, and PAm gel matrix was represented as black. In this way, the signal to noise ratio for tracking the diffusion of copper ions was improved.

#### Counter-Diffusion Experiments

Mold was designed to be capable of holding a 1 cm column of PAm hydrogel without any inner electrolyte. The molds were designed similarly to 1D gel, i.e., a spacer of height 0.2 cm is sandwiched between two plexiglass pieces of the same dimensions. The spacer’s one end closed while the other end remains open. The plexiglass piece at the top has a cube-like opening of dimensions 0.3 cm × 0.3 cm into the spacer. Then, 0.73 g of AA, 0.0015 g of BIS, 0.01 g of KPS were dissolved in 4.8 mL of water. The solution is ultrasonicated for 2 min and then, 5 μL of TEMED was added. The solution was then transferred to the mold through an open end and allowed gelation process to complete for 24 h. After that, 1.0 M CuCl_2_ was introduced through the open end of the mold. The desired concentration of potassium chromate was inserted through the cube-like opening on the top Plexiglas piece. A small drop of oil is added on top of CuCl_2_ and K_2_CrO_4_ solution inside the mold to reduce the evaporation of the solutions.

#### Time Law

The time law was determined by loading the stack of images into ImageJ. Each frame in the image stack represents an image taken at a 5 min interval. The PC between the gel and the outer electrolyte was chosen as a reference point for all the images. The position of the bands was determined by stretching the line from PC to the middle of the band. The time was decided as the point where ring starts to form. A plot was plotted regarding the relationship between the position of the band and the square root of time.

### Determination of Copper(II) Chromate’s Solubility Product in Water

#### Synthesis of Copper(II) Chromate

First, 6.7 g of copper(II) chloride and 9.7 g of potassium chromate were dissolved separately in 250 mL of water each, and then ultrasonicated until complete dissolution. Copper(II) chloride solution was added slowly to the potassium chromate solution. The reaction mixture was stirred 12 h. Then, the precipitate was filtered, washed with water (8 × 200 mL) and methanol (8 × 200 mL), and dried under vacuum.

#### Copper(II) Chromate’s Solubility in H_2_O

Two milliliters of water was added to 25 mg of copper(II) chromate, and the solution was stirred for 12 h at a desired temperature. After that, the solution was transferred to syringe (preheated at a desired temperature), and filtered using a syringe filter (preheated at a desired temperature). Then, 0.5 mL of the filtrate was diluted to 10 mL with water. The solution was then analyzed with UV–vis spectroscopy between 365–370 nm to obtain the concentration of chromate ions. A calibration plot for UV–vis spectroscopy was obtained from 0.01, 0.025, 0.05, 0.075, 0.1, and 0.25 mM solutions of potassium chromate prepared by appropriate dilutions.

#### SEM and Energy Dispersive X-ray Analyses

Hydrogel with LPs formed at a desired temperature is placed in a glass flask. Vacuum is applied and the glass flask is transferred to liquid nitrogen bath for 30 min. Then, frozen hydrogels are cut in half using a steel blade and dried under vacuum at room temperature for 24 h.

The surface morphology of gel samples was imaged and analyzed with a Quanta 200F model SEM with an accelerating voltage of 10 kV. Samples were coated with Au-Pd. The particle sizes were determined using digital image analysis ImageJ software.

## Supporting Information Available

Studying the effect of CuCl_2_, K_2_CrO_4_, and BIS concentration on the LP formation; plexiglass mold designs for the preparation of 1D, 2D, and 3D PAm gels; protocol for the determination of spacing coefficient; numerical simulations of precipitation patterns formed at desired temperature experiments and calculated spacing coefficients; band formation system with: different transition times, different ramping rate of temperature change; monitoring the diffusion of copper ions in PAm hydrogel at indicated temperature; counter diffusion experiments; variance between the time and position of the pattern evolution; determination of copper(II) chromate’s solubility product in water; discussion on the relationship between reaction coefficient, diffusion coefficient, and precipitation threshold, pushed and pulled fronts; histograms representing particle size distributions retracted from SEM micrographs of indicated bands and given temperatures; SEM–EDX analysis of depletion band and band region; temperature ramping rate experiments (PDF)
Time evolution of the precipitation patterns and formation of LP at 20 °C formed in PAm hydrogels. 1 M CuCl_2_ solution applied at the PC is let to diffuse (from left to right in the movie) into gel strips containing 0.01 M K_2_CrO_4_ (AVI)
Time evolution of the precipitation patterns and formation of LP at 30 °C formed in PAm hydrogels. 1 M CuCl_2_ solution applied at the PC is let to diffuse (from left to right in the movie) into gel strips containing 0.01 M K_2_CrO_4_ (AVI)
Time evolution of the precipitation patterns and formation of LP at 40 °C formed in PAm hydrogels. 1 M CuCl_2_ solution applied at the PC is let to diffuse (from left to right in the movie) into gel strips containing 0.01 M K_2_CrO_4_ (AVI)
Time evolution of the precipitation patterns and formation of LP at 50 °C formed in PAm hydrogels. 1 M CuCl_2_ solution applied at the PC is let to diffuse (from left to right in the movie) into gel strips containing 0.01 M K_2_CrO_4_ (AVI)
Time evolution of the precipitation patterns and formation of LP at 60 °C formed in PAm hydrogels. 1 M CuCl_2_ solution applied at the PC is let to diffuse (from left to right in the movie) into gel strips containing 0.01 M K_2_CrO_4_ (AVI)

## Supplementary Material

am1c20640_si_001.pdf

am1c20640_si_002.avi

am1c20640_si_003.avi

am1c20640_si_004.avi

am1c20640_si_005.avi

am1c20640_si_006.avi
