# Supplementary material for: Chemical Tracking of Temperature by Concurrent Periodic Precipitation Pattern Formation in Polyacrylamide Gels
Source: ACS Appl Mater Interfaces. 2022 Jan 20;14(5):7252–60. doi: 10.1021/acsami.1c20640 (PMC8895381; doi:10.1021/acsami.1c20640)
Supplement: Supplementary file 1 — am1c20640_si_001.pdf [file am1c20640_si_001.pdf]

Supporting Information

Chemical Tracking of Temperature by Concurrent Periodic Precipitation Pattern Formation in Polyacrylamide Gels

Muhammad Turab Ali Khan,[a,‡] *Joanna Kwiczak-Yiğitbaşı,*[a,‡] *Pedram Tootoonchian,*[a] *Mohammad Morsali,*[a] *Istvan Lagzi,**[b] *Bilge Baytekin,**[a,c]

[a] Chemistry Department, Bilkent University, Ankara

[b] Department of Physics and BME-MTA Condensed Matter Physics Research Group, Budapest University of Technology and Economics, Budapest H-1111, Hungary

[c] UNAM, Bilkent University, Ankara 06800

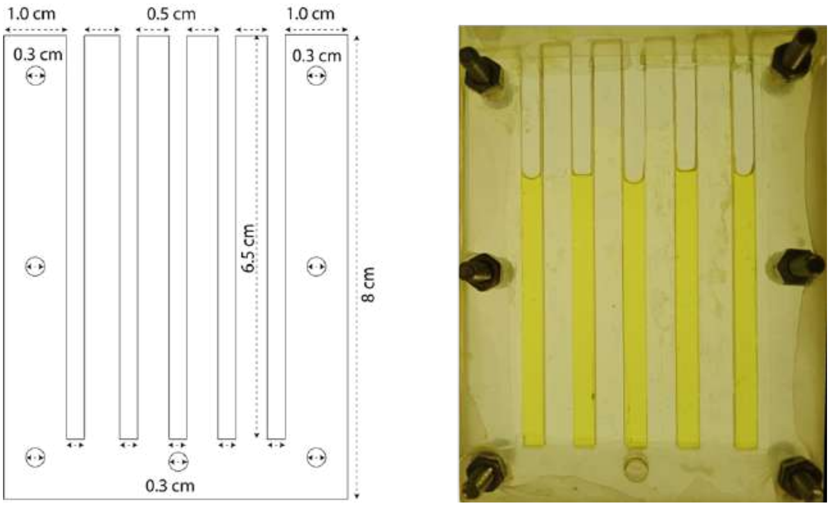

Figure S1. The left side shows the dimensions of the spacer used for 1 D gels. The spacer was sandwiched between two Plexiglas pieces with the same dimensions, using screws. On the right, the gel samples in 1D gel samples without the addition of the outer electrolyte are shown.

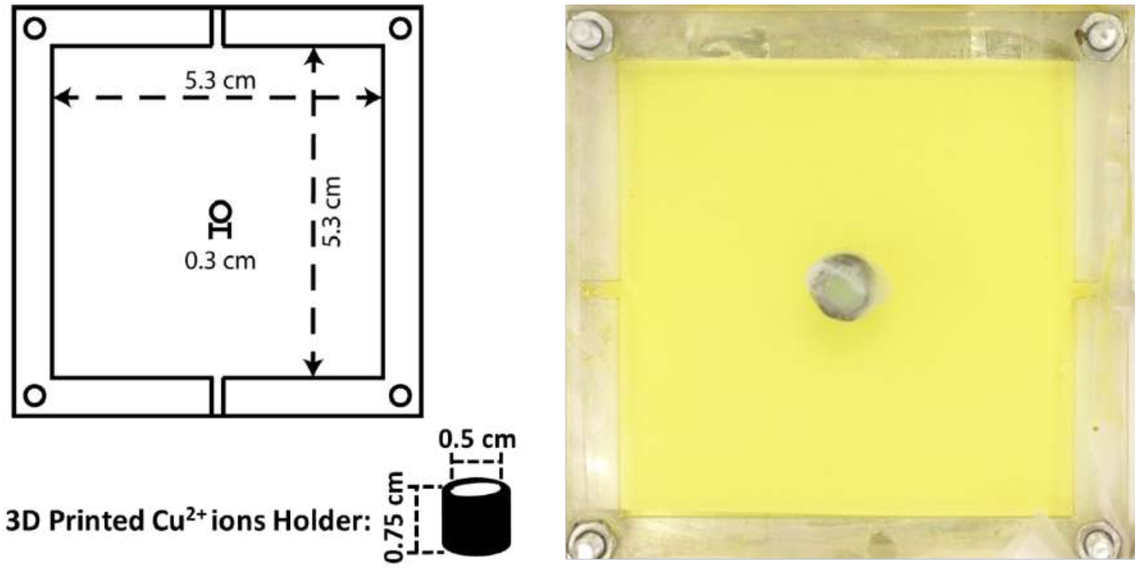

Figure S2. The left side shows the dimensions of the mold to prepare 2D gel sheets. The top side has a hole of the diameter of 0.3 cm. The 3D printed holder for introducing Cu^{2+} was attached on top of the hole using Superglue. On the right, the gel samples in 2D gel samples with the addition of the outer electrolyte are shown.

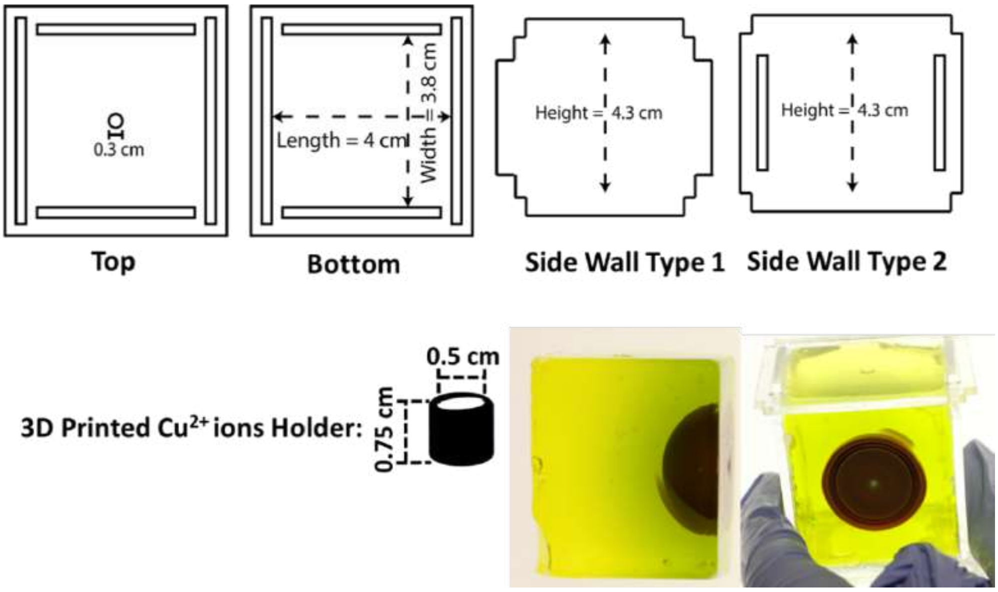

Figure S3. Top images show the dimensions for each of the sides of the cubical box. The pieces were fitted into one another and the edges were sealed from outside using Ecoflex 0030. The 3D printed Cu^{2+} holder was attached to the top face of the cube. 3D samples with patterns formed inside the cubical box are shown at the bottom right.

[BIS]	[$CuCl_2$]	[K_2CrO_4]	Appearance of Liesegang patterns
0.004	0.1	0.005	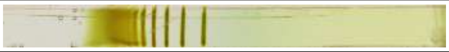
		0.010	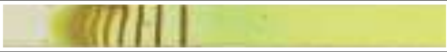
		0.015	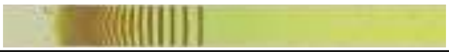
**0.004**	**0.5**	**0.005**	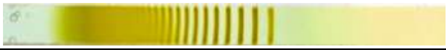
		**0.010**	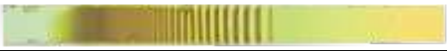
		0.015	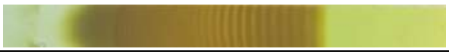
**0.004**	**1.0**	0.005	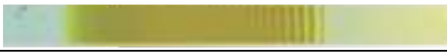
		**0.010**	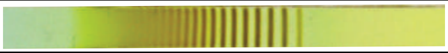
		0.015	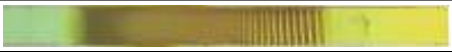
0.004	1.5	0.005	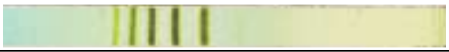
		0.010	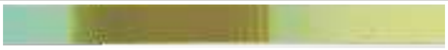
		0.015	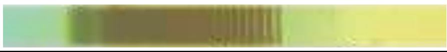
**0.0015**	1.0	0.01	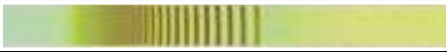
**0.010**			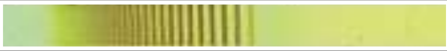

1 cm

Figure S4. Studying the effect of the concentration of crosslinker, outer and inner electrolyte on the formation of LPs. Based on the Liesegang pattern appearances concentration of $CuCl_2$, K_2CrO_4 should be in the range of 0.5-1.0 M, and 0.005-0.010 M, respectively. Satisfactory formation of LPs was also achieved when BIS concentration was in the range of 0.0015-0.01 M.

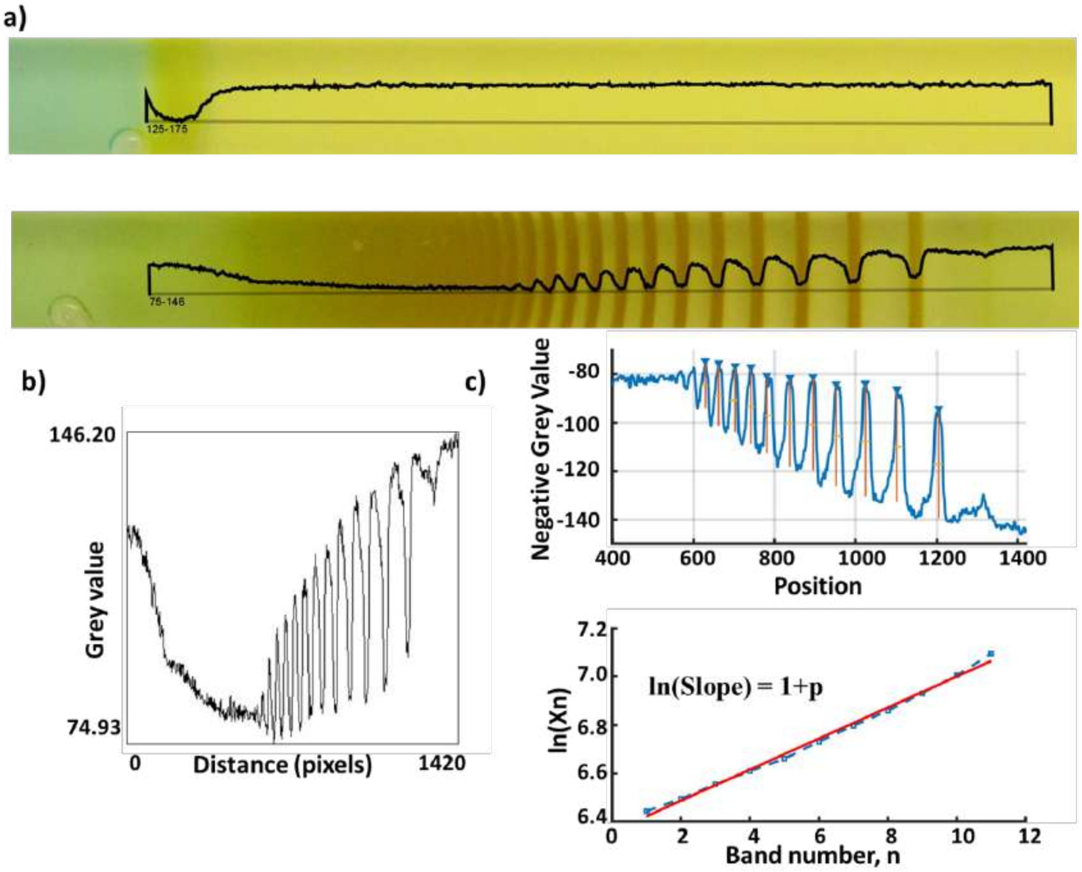

Figure S5. a) The gray value profile (black line) is plotted for the first photo taken during the experiments and the last photo. The bands are appear as dips in the Gray-value. b) The Gray-value plot over distance. c) Negative of Gray-value is plotted against distance i.e. position in Matlab. The bands appear as peaks and using ‘Findpeak’ the logarithm of position of bands against band number. The logarithm of slope gives us 1+*p* i.e. the spacing coefficient.

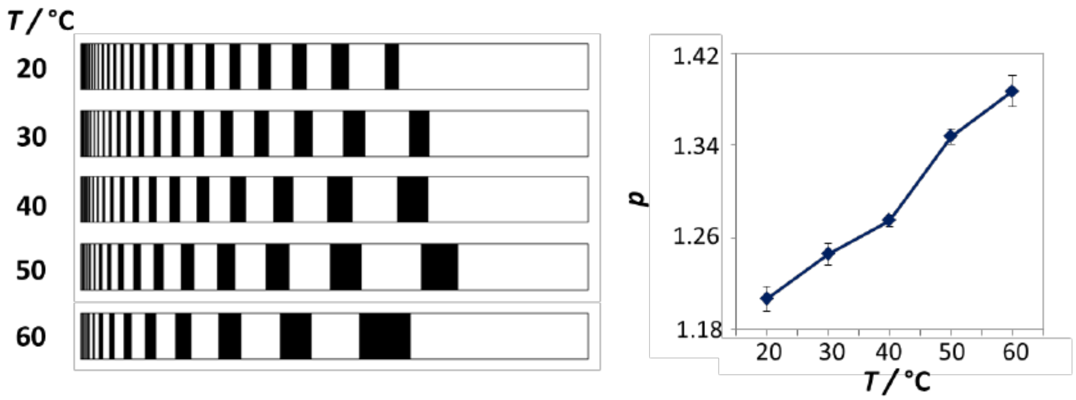

Figure S6. The results of numerical simulations of precipitation patterns formed at desired temperature and calculated spacing coefficient.

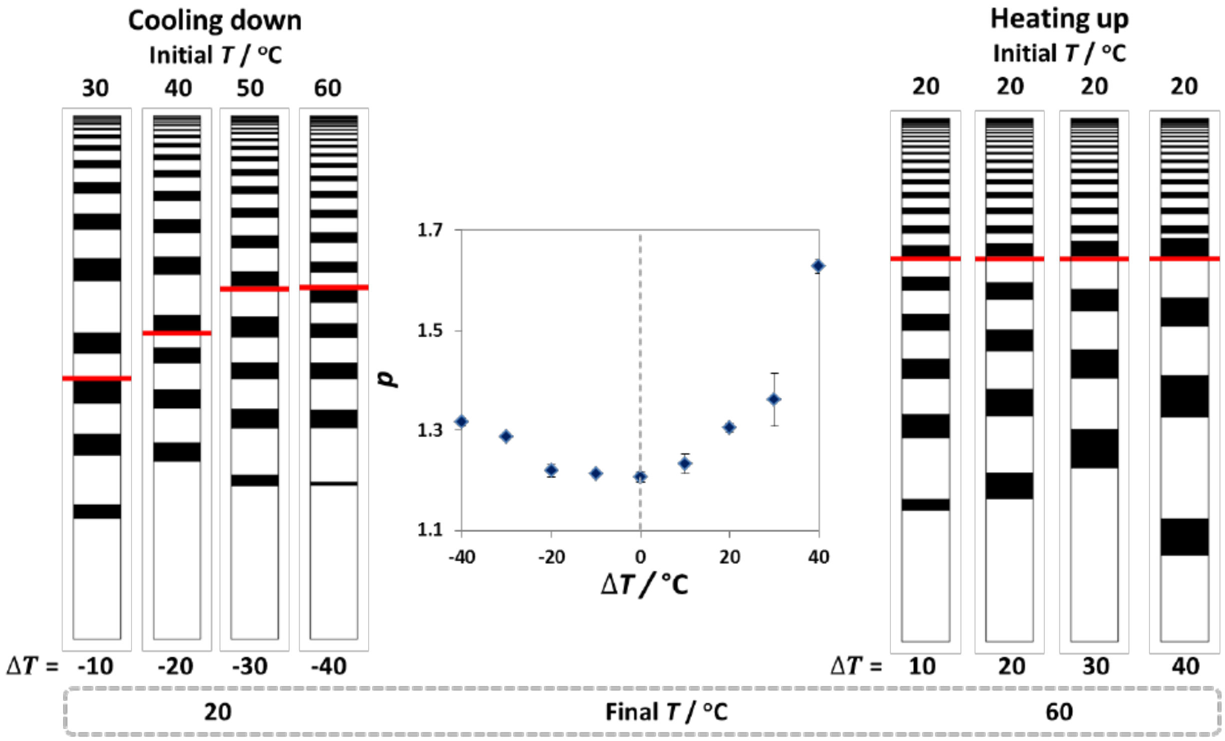

Figure S7. The results of calculated spacing coefficient and numerical simulations of precipitation patterns formed when the temperature was decreased from 30, 40, 50, and 60 °C to 20 °C or increased from 20 °C to 30, 40, 50, and 60 °C.

$[K_2CrO_4]$	$[CuCl_2]$	Appearance of Liesegang patterns	p value
0.01	0.1	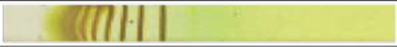	1.356 ± 0.005
	0.5	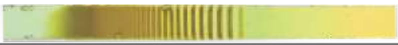	1.085 ± 0.002
	1.0	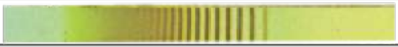	1.048 ± 0.010

1 cm

Figure S8. When the concentration of outer electrolyte decreases, the spacing coefficient values increase. $CuCl_2$ solution is let to diffuse (from left to right in the photos) into gel strips containing 0.01 M K_2CrO_4 at 20 °C. Error bars correspond to standard deviations from five independent experiments.

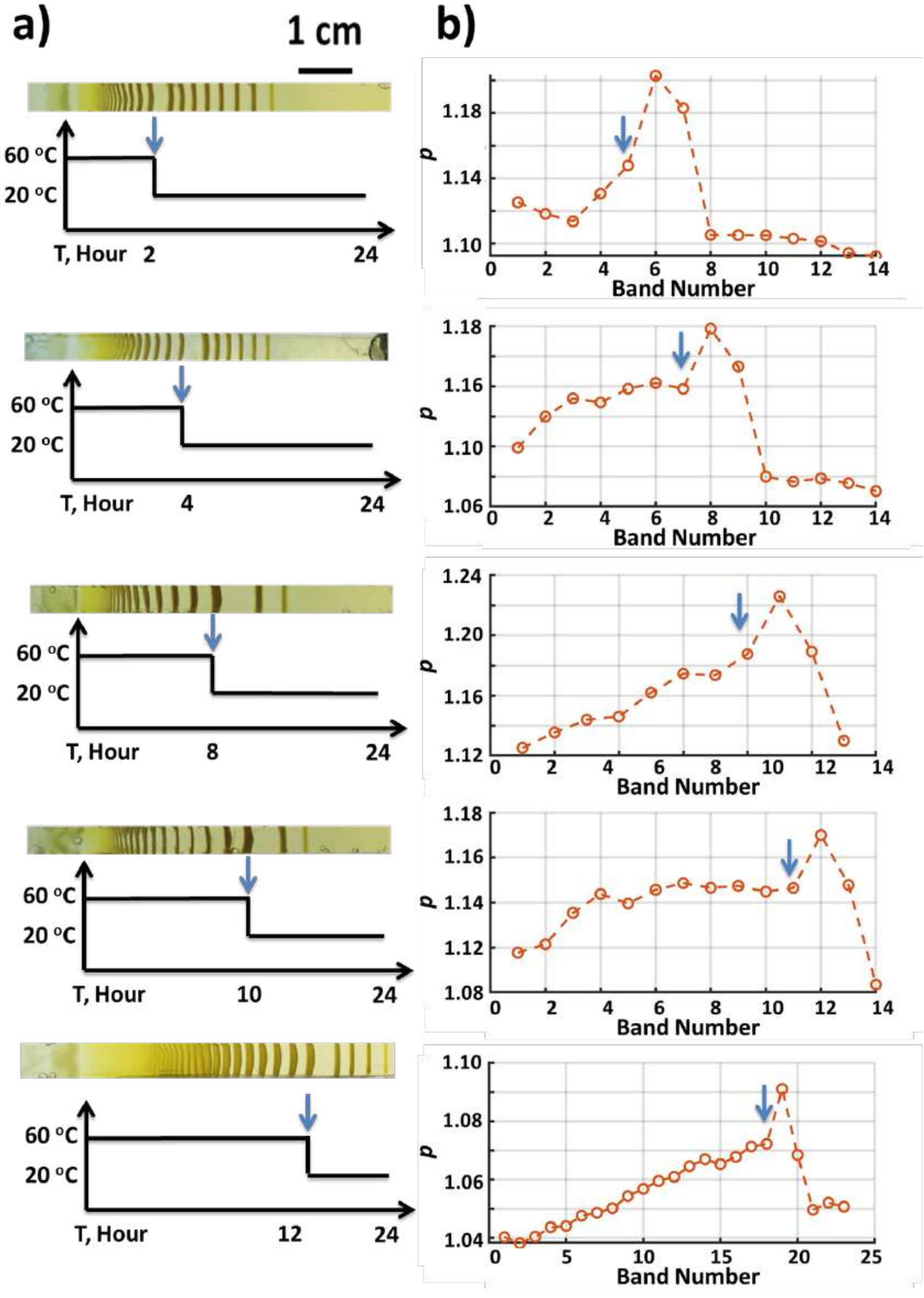

Figure S9. Band forming system with different transition down times. a) From top to bottom, the samples were kept for 2 h, 4 h, 8 h, 10 h and 12 h at 60 °C following the decrease in temperature to 20 °C till the 24th h. b) The variation in the spacing coefficient of the bands along the spatial coordinate.

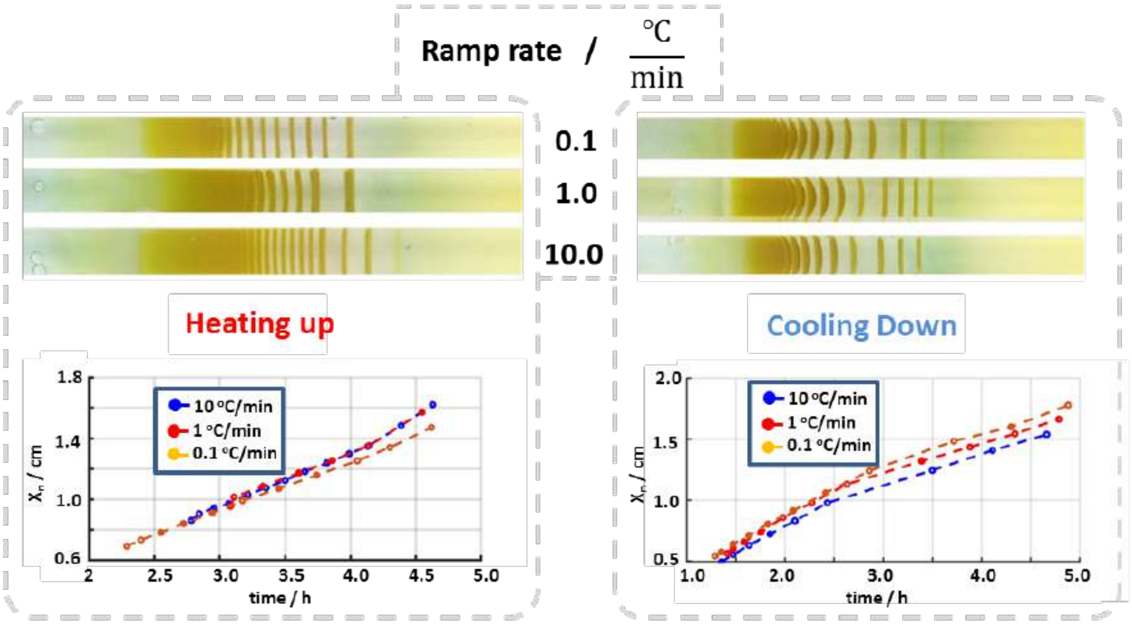

Figure S10. The variation in the time evolution of patterns in samples with difference in ramping rate of temperature change. Samples are placed at 20 °C for 17 hours and then temperature is increased to 50 °C at ramp rates of 0.1, 1 and 10 °C/min and then temperature is fixed at 50 °C till 24th hour of the experiment (right). Samples are placed at 50 °C for 7 hours and then temperature is decreased to 20 °C at ramp rates of 0.1, 1 and 10 °C/min and then temperature is fixated at 20 °C till 24th hour of the experiment (left). Space-time plots show the change in pattern evolution over time for different initial conditions and ramps.

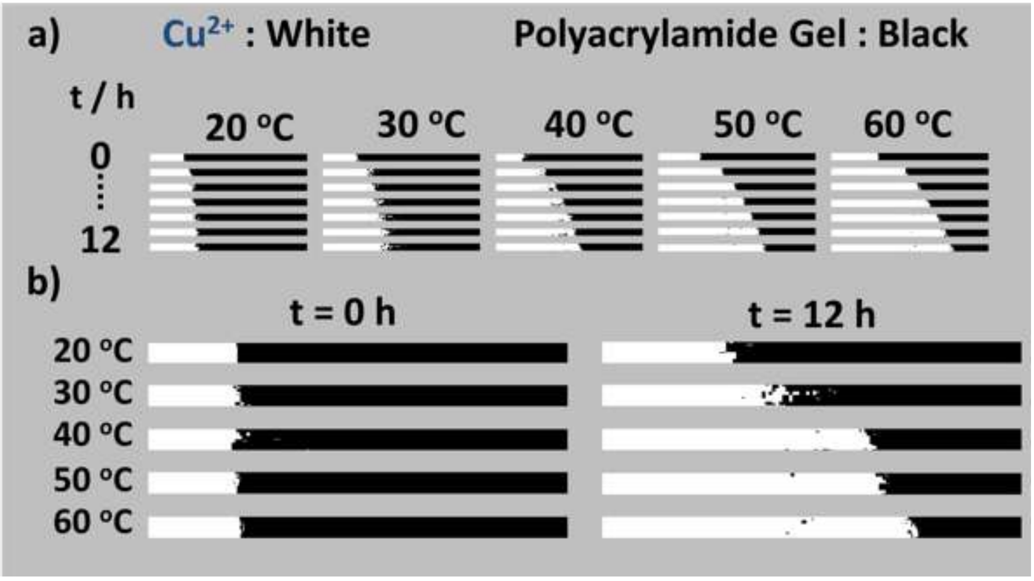

Figure S11. Diffusion of copper ions in polyacrylamide hydrogel without inner electrolyte (K_2CrO_4) was monitored at different temperatures. Copper ions are represented in white, while polyacrylamide hydrogel is represented in black. a) Diffusion of copper ions at each temperature from 0 to 12 h with time interval between each frame to be of 2 hours. b) Comparison of how the diffusion in a constant time interval differs with each temperature. *t* = 0 h the first frame and *t* = 12 h is the last frame of the time lapse for each of the temperatures.

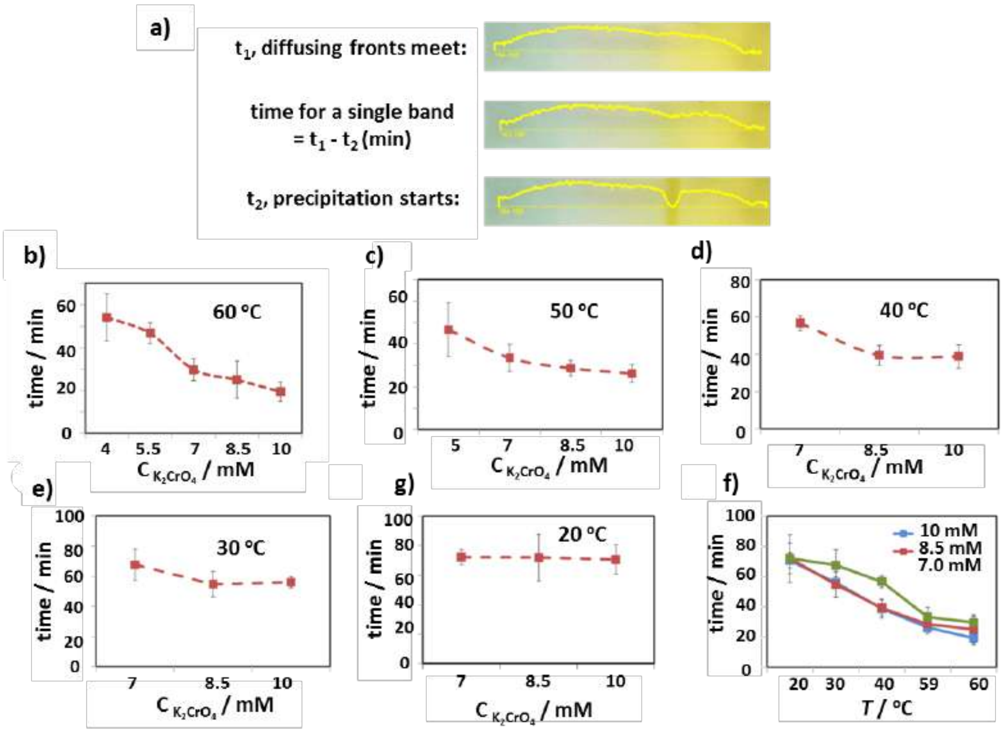

Figure S12. a) The time at which the fronts meet is denoted as t_1, and the time for the precipitation band to appear visually is demonstrated as t_2. The time for formation of the single precipitation band is the difference between t_2 - t_1. The time for the formation of a single precipitation band at different potassium chromate concentrations at b) 60 °C, c) 50 °C, d) 40 °C, e) 30 °C, f) 20 °C. g) The time for the appearance of the band versus temperature at a particular concentration.

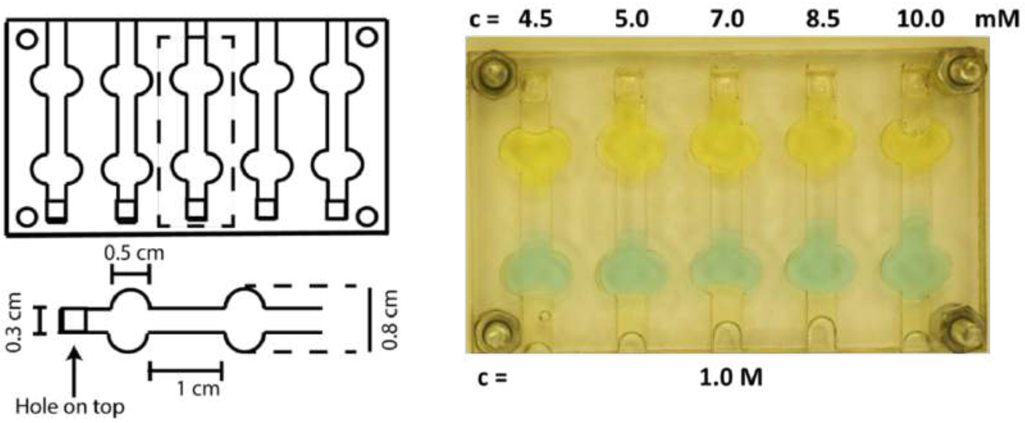

Figure S13. On the left, the dimensions of the mold to prepare a 1 cm × 0.3 × 0.2 cm PAM gel column is shown. The dimensions of each reservoir of electrolytes are 0.5 × 0.8 × 0.2 cm. Mold filled with the copper(II) chloride and potassium chromate solutions on each side of 1 cm polyacrylamide gel stripe (right).

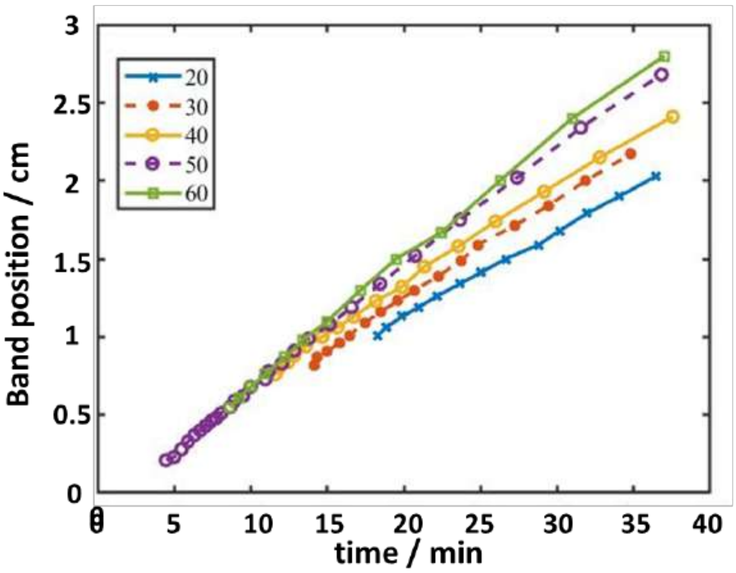

Figure S14. The variance between the time and position of the pattern evolution.

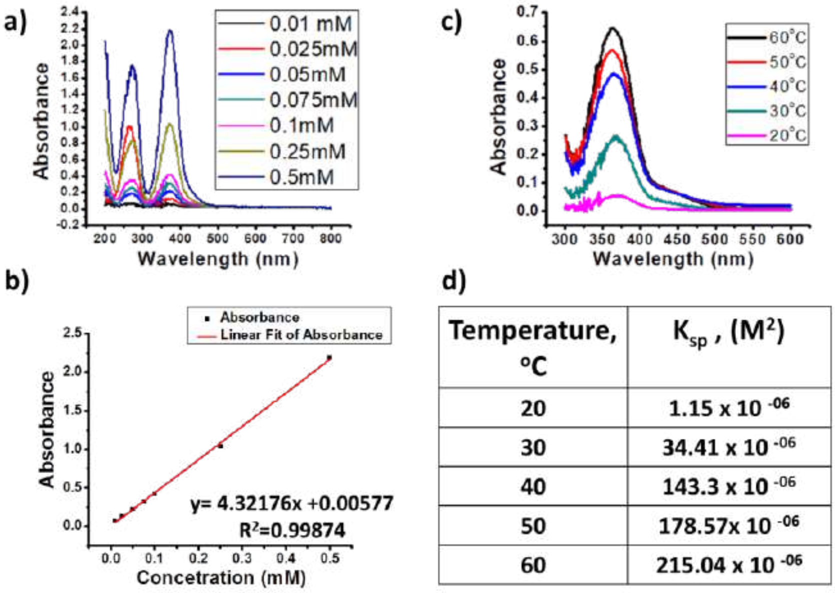

Temperature, °C	K_{sp} , (M^2)
20	1.15 x 10 $^{-06}$
30	34.41 x 10 $^{-06}$
40	143.3 x 10 $^{-06}$
50	178.57x 10 $^{-06}$
60	215.04 x 10 $^{-06}$

Figure S15. a) UV-Vis absorption spectra of 0.01, 0.025, 0.05, 0.075, 0.1 and 0.25 mM. b) Calibration plot for determining the concentration of chromate ions, plotted from the data obtained in (a) at 365-370 nm. c) UV-Vis absorption spectra of samples from copper chromate solutions at different. d) The variance in solubility product as a function of temperature is shown in table.

Further discussions on the relationship between reaction coefficient (k_p), diffusion coefficient (k_d), and precipitation threshold (k_{sp}) and pushed and pulled fronts

The evolution of the patterns when subjected to a change in temperature can be understood using the triangular relationship between reaction coefficient (k_p), diffusion coefficient (k_d), and precipitation threshold (k_{sp}) and pushed and pulled fronts. Firstly, let's dissect a theoretical approach proposed by Antal et al. for such forced manipulation of an LP system. In their work, they suggested a diffusive guiding field, originating from one end could result in inverse and equidistant banding. Guiding field could be a temperature gradient such that the reaction front gets cooled down as it propagates through the system. As a result, the front propagates slowly as it moves along the temperature field and after certain point patterns form with decreasing distances between them (inverse banding). This is an example of coherent patterns forming behind a pulled reaction front. In our experimental conditions, a pulled reaction front occurs when temperature is lowered at a specific time (Figure S9) and additionally, we also pull the reaction front at a specific rate as well (Figures S10, S21, S22). Even though our system lacks a temperature gradient across the length of the gel, our results indicate manipulation of patterns and localized inverse banding. This is observed in terms of the decrease in the spacing coefficient (Figure 2). Thus, pulling a front means lowering the diffusion coefficient of copper ions and thus, slowing down the flux of copper ions. At the same instant, the solubility product of copper(II) chromate decreases and the precipitation threshold is lowered, along with, the rate constant. With all three integral components of the equation lowered, LPs transition to a lower temperature regime. A halt in the supply of the copper ions from the source results in a wider depletion zone beyond the last pre-transition band formed. As the front moves slowly and lowered reaction rate, results in lower production of colloids, their dissolution, re-precipitation and aggregation. With the lowered precipitation

threshold, it is expected to see a continuous precipitation beyond pre-transition patterns and before post-transition patterns; however, such a visual change is not observed. In this scenario, k_p, and k_d dominate k_{sp}. Instead, a wide band appears as the first post-transition band. The width of this band indicates aggregation of a higher number of colloids to form a band. Additionally, this wider band requires higher time interval to form, and thus, lowers the slope of space-time plot (Figure S10). This increased time interval refers to the relaxation time due to a pulled front.

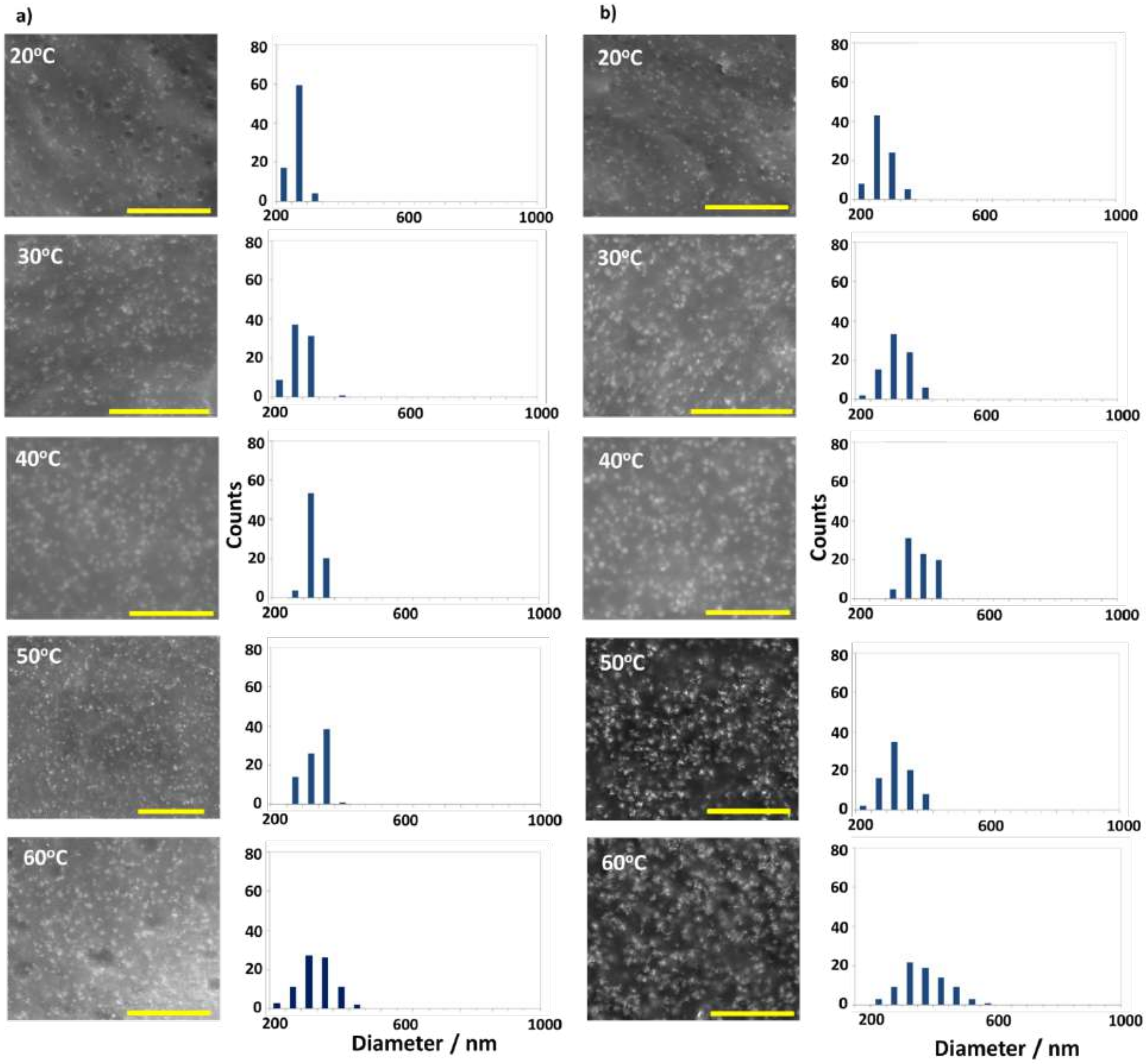

Figure S16. Histograms presenting particle size distributions (right) retracted from SEM micrographs (left) of band a) 1, and b) 5 formed at given temperature. The particle sizes increase with increasing temperature and also this increasing trend is observed moving from band 1 to 5. The scale bar is 5 μm.

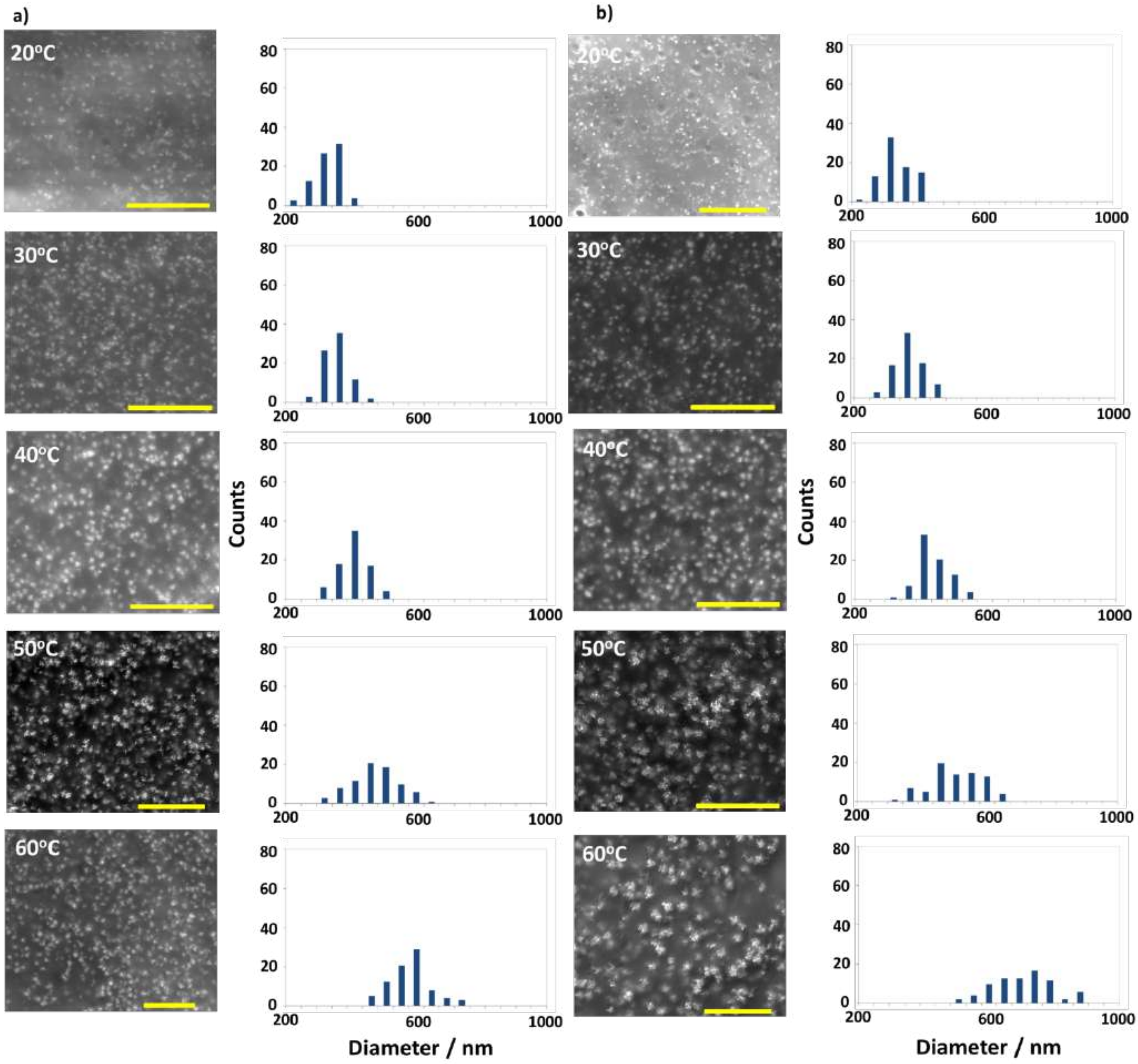

Figure S17. Histograms presenting particle size distributions (right) retracted from SEM micrographs (left) of band a) 7, and b) 9 formed at given temperature. The particle sizes increase with increasing temperature and also this increasing trend is observed moving from band 7 to 9. The scale bar is 5 μm.

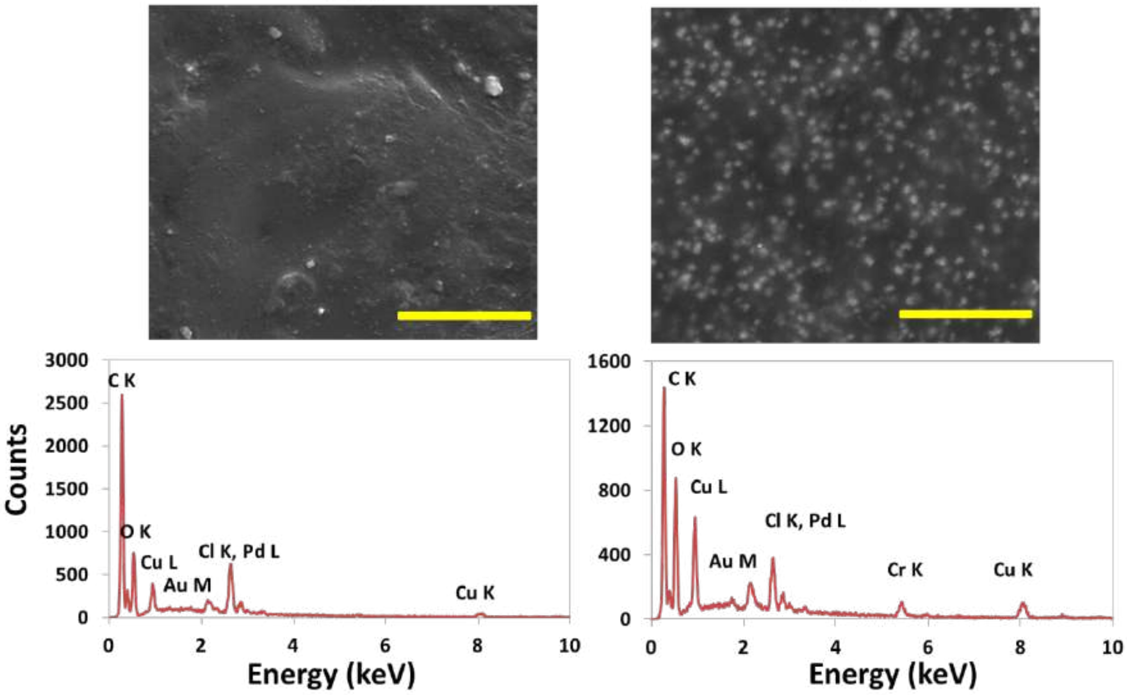

Figure S18. SEM micrographs showing the difference between a depletion band (top left) and band region (top right). The scale bar is 5 μm. The elemental analysis using EDX shows difference between band (bottom right) and non-band region (bottom left), with the occurrence of Cr peak in band region.

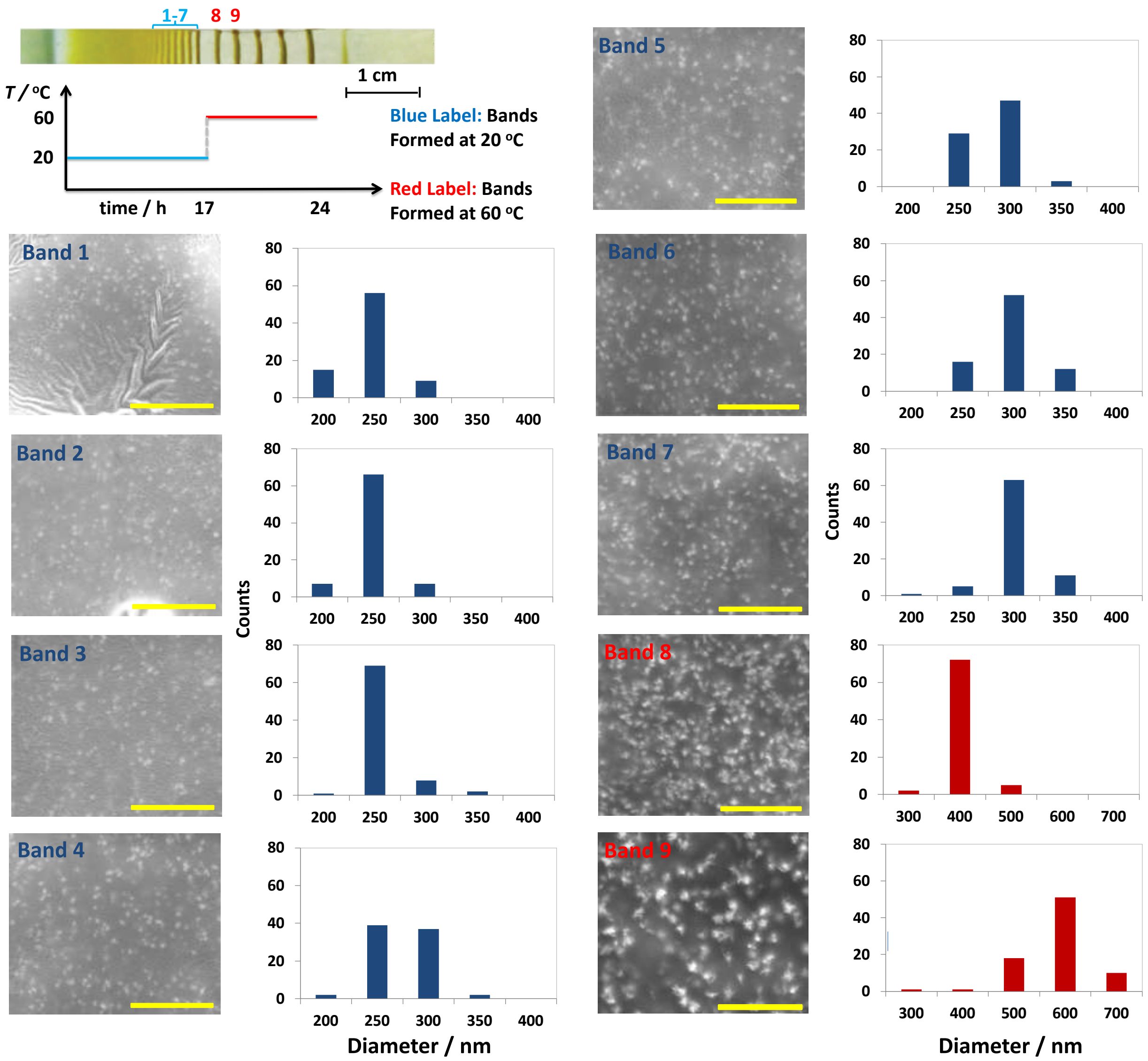

Figure S19. Histograms presenting particle size distributions retracted from SEM micrographs of bands 1 to 9 formed at 20 °C (bands: 1 – 7) and 60 °C (bands: 8, 9). When the temperature was increased from 20 °C to 60 °C the particle sizes increased significantly. The scale bar is 5 μm.

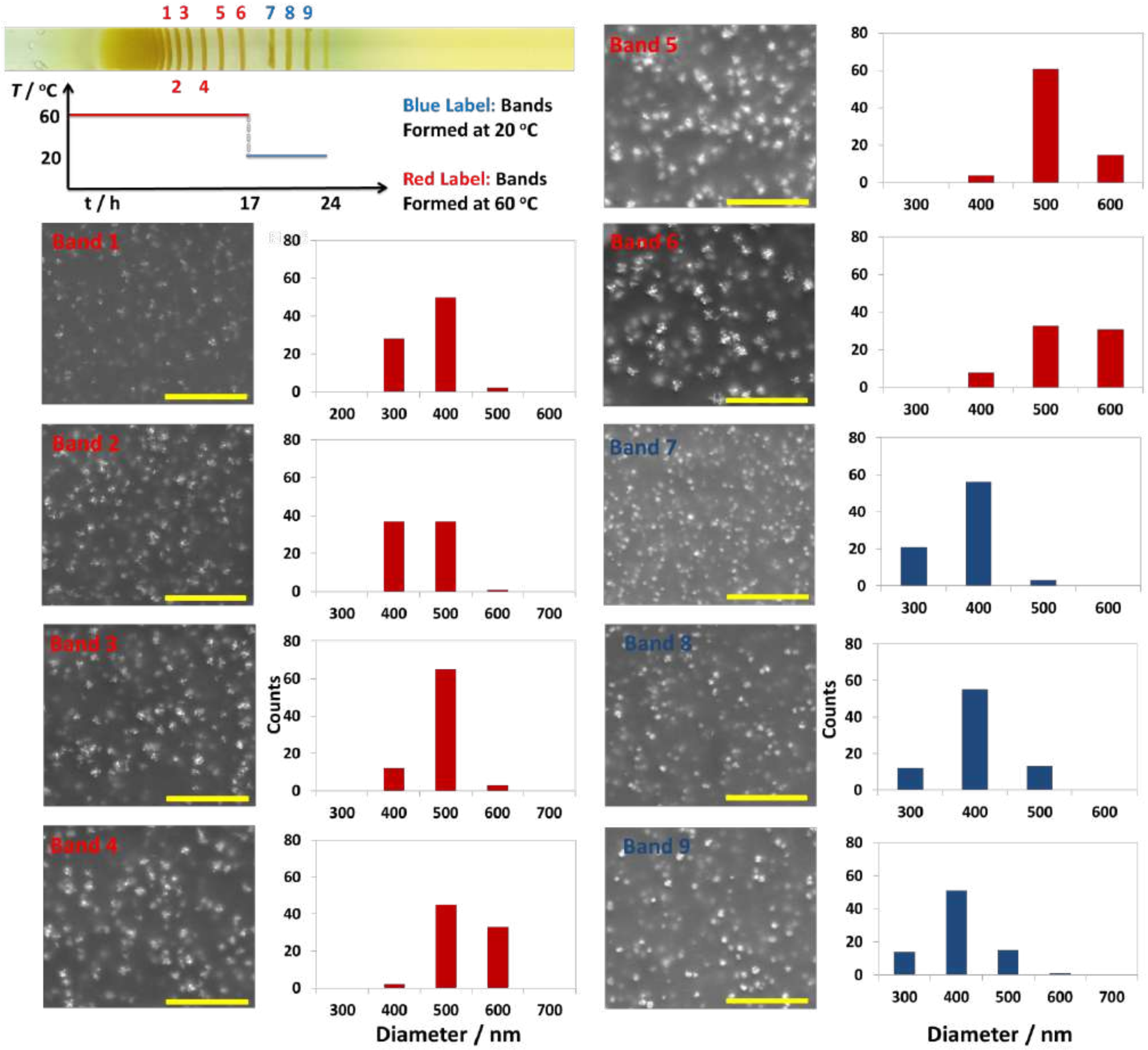

Figure S20. Histograms presenting particle size distributions retracted from SEM micrographs of bands 1 to 9 formed at 60 °C (bands: 1 – 6) and 20 °C (bands: 7 – 9). When the temperature was decreased from 60 °C to 20 °C the particle sizes decreased significantly. The scale bar is 5 μm.

Temperature Ramping Rate Experiments

In case of ramping temperature down, the relaxation time is higher for a sudden temperature change (10 °C/min) compared to a gradual change in the system's temperature (0.1 °C/min). Thus, the slopes of cooling down LP system (Figure S21) demonstrate such trend. Later on, the spacing between post-transition bands starts to increase, however, the increase is not comparable to pre-transition patterns formed at higher temperatures. Thus, the system relaxes after the perturbation in the form of a pulled front and returns to its normal characteristics. Perturbing the system by heating up results in an increase in k_p, k_d and k_{sp} and a pushed front (Figure S22). In case of the pushed front, the flux of the copper ions from the source increases. The reaction rate constant indicates that all parts of the complex chain of events leading to periodic precipitation patterns i.e. formations of colloids, their dissolution, re-precipitation as bigger colloids and aggregation into a periodic band structure occur at a higher rate (Figure S22). Thus, bands start to occur immediately upon the rise in temperature. At this point the k_p and k_d dominate k_{sp}, because if k_{sp} had dominated an immediate zone would not be observed and wider depletion zone would have been observed. The raised solubility product adds to the fact that wider depletion zones are observed post-transition patterns. With a pushed front, the system does not undergo a relaxation period and that is why the slopes of the space-time plots for post-transition patterns increase. Similarly, the rate at which temperature changes effects the extent of a pushed front and thus faster moving fronts (10 °C/min) will provide with steeper slopes (Figure S22).

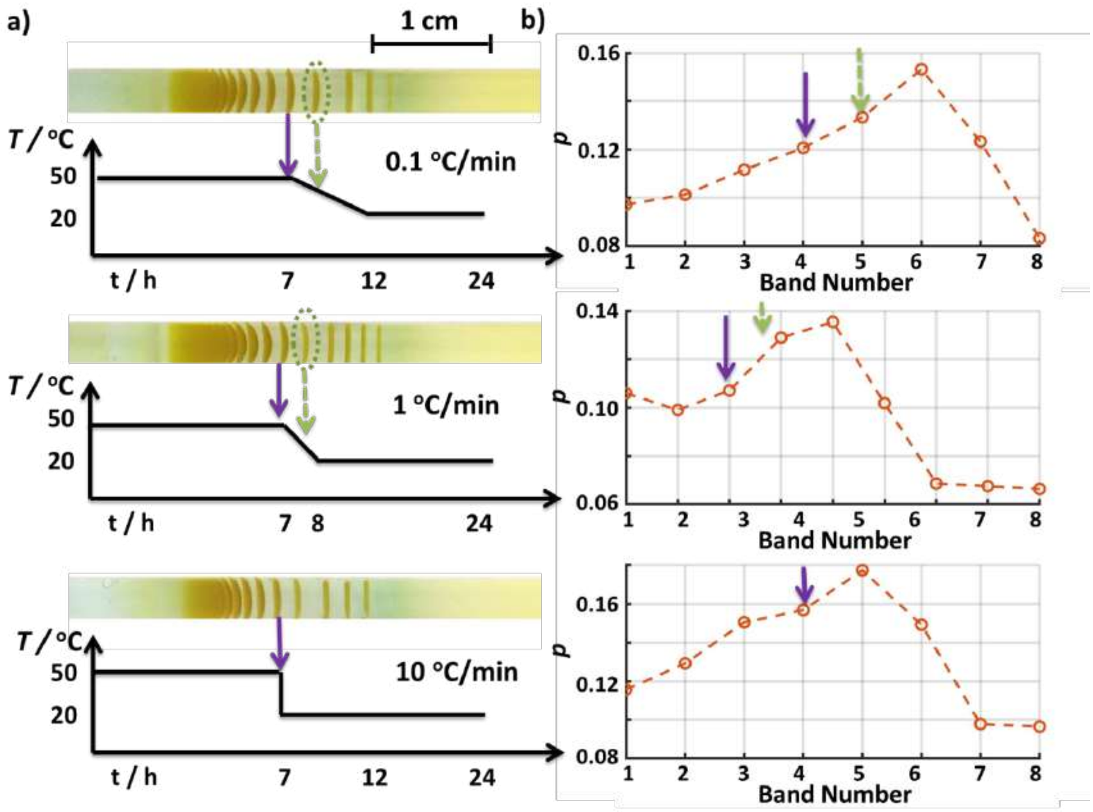

Figure S21. a) Changing the temperature from 50 °C to 20 °C at indicated ramp rate. b) The spacing coefficient variation with respect to the band number at indicated ramp rate. Blue arrow indicates the point of transition and the green arrow represents the pattern formed during the transition period.

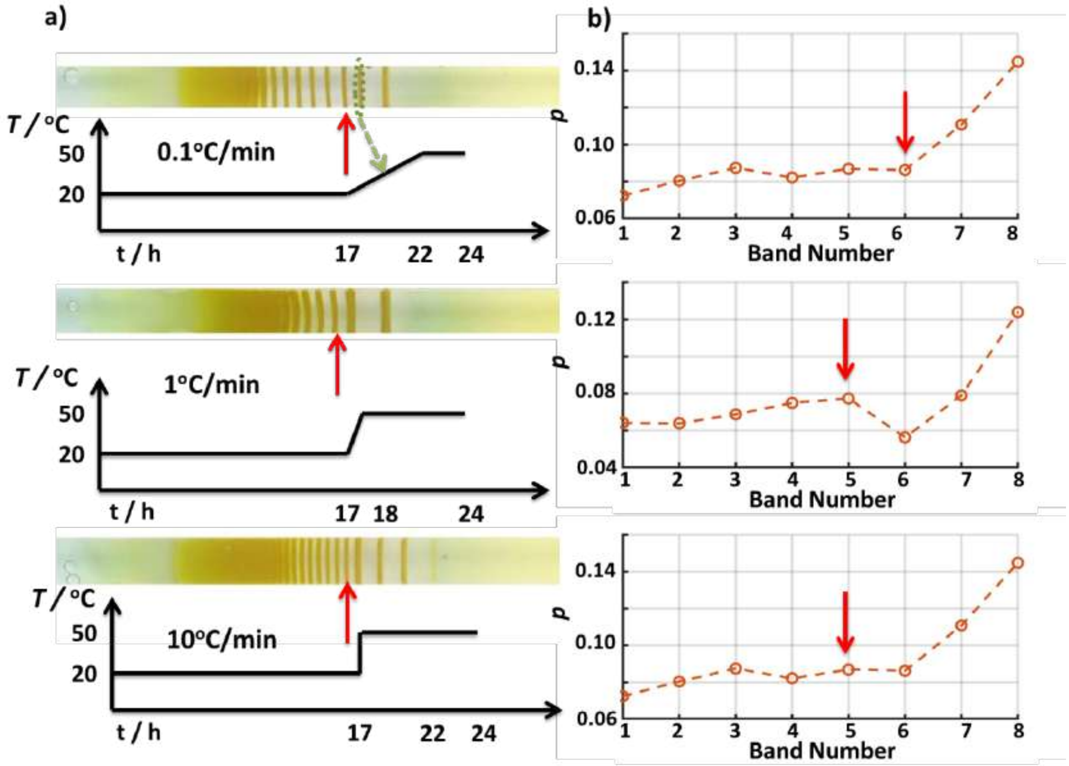

Figure S22. a) Changing the temperature from 20 °C to 50 °C at indicated ramp rate. b) The spacing coefficient variation with respect to the band number at indicated ramp rate. Red arrow indicates the point of transition and the green arrow represents the band formed during the transition period.
